# The evolution of protostome GATA factors: Molecular phylogenetics, synteny, and intron/exon structure reveal orthologous relationships

**DOI:** 10.1186/1471-2148-8-112

**Published:** 2008-04-15

**Authors:** William Q Gillis, Bruce A Bowerman, Stephan Q Schneider

**Affiliations:** 1 Institute of Molecular Biology, University of Oregon, 1229 University of Oregon, Eugene, OR 97403, USA

## Abstract

**Background:**

Invertebrate and vertebrate GATA transcription factors play important roles in ectoderm and mesendoderm development, as well as in cardiovascular and blood cell fate specification. However, the assignment of evolutionarily conserved roles to GATA homologs requires a detailed framework of orthologous relationships. Although two distinct classes, GATA123 and GATA456, have been unambiguously recognized among deuterostome GATA genes, it has been difficult to resolve exact orthologous relationships among protostome homologs. Protostome GATA genes are often present in multiple copies within any one genome, and rapidly evolving gene sequences have obscured orthology among arthropod and nematode GATA homologs. In addition, a lack of taxonomic sampling has prevented a stepwise reconstruction of protostome GATA gene family evolution.

**Results:**

We have identified the complete GATA complement (53 genes) from a diverse sampling of protostome genomes, including six arthropods, three lophotrochozoans, and two nematodes. Reciprocal best hit BLAST analysis suggested orthology of these GATA genes to either the ancestral bilaterian GATA123 or the GATA456 class. Using molecular phylogenetic analyses of gene sequences, together with conserved synteny and comparisons of intron/exon structure, we inferred the evolutionary relationships among these 53 protostome GATA homologs. In particular, we resolved the orthology and evolutionary birth order of all arthropod GATA homologs including the highly divergent *Drosophila *GATA genes.

**Conclusion:**

Our combined analyses confirm that all protostome GATA transcription factor genes are members of either the GATA123 or GATA456 class, and indicate that there have been multiple protostome-specific duplications of GATA456 homologs. Three GATA456 genes exhibit linkage in multiple protostome species, suggesting that this gene cluster arose by tandem duplications from an ancestral GATA456 gene. Within arthropods this GATA456 cluster appears orthologous and widely conserved. Furthermore, the intron/exon structures of the arthropod GATA456 orthologs suggest a distinct order of gene duplication events. At present, however, the evolutionary relationship to similarly linked GATA456 paralogs in lophotrochozoans remains unclear. Our study shows how sampling of additional genomic data, especially from less derived and interspersed protostome taxa, can be used to resolve the orthologous relationships within more divergent gene families.

## Background

GATA transcription factors perform conserved and essential roles during animal development, including germ layer specification, hematopoiesis, and cardiogenesis [1]. Nevertheless, homologs in the GATA gene family have undergone significant divergence in both sequence and gene number in different animal phyla, making it difficult to resolve orthologous relationships of individual family members [2,3]. For example, the number of GATA paralogs – homologs within an individual genome – varies substantially between protostomes and deuterostomes. Most vertebrate genomes possess six GATA paralogs, whereas the fruitfly *Drosophila melanogaster *has only five and the nematode/roundworm *Caenorhabditis elegans *eleven. Reconstructing the evolution and the ancestral developmental roles of these genes requires a framework of orthologous relationships among GATA homologs.

Previous studies have identified two classes of GATA homologs within deuterostomes [2,3]. Basal invertebrate deuterostomes, including echinoderms, urochordates, and cephalochordates, possess only single GATA123 and GATA456 orthologs. Most vertebrates possess three paralogs from each class, likely from two whole genome duplication events that occurred during the evolution of jawed vertebrates. Within the three vertebrate GATA123 paralogs, the vertebrate GATA-2 and -3 genes are more closely related to each other than to the GATA-1 gene. Likewise, the vertebrate GATA-4 and -6 genes are both more closely related to each other than to the GATA-5 gene [3]. Thus two genome duplications, together with the losses of one GATA-1 like paralog and one GATA-5 like paralog, can account for the number of genes in each vertebrate GATA class.

While the evolution of GATA factors within the deeper branches of the deuterostome phylogeny is well understood, it has been more difficult to reconstruct the evolution of protostome GATA factors. We recently published data suggesting that the last common protostome/deuterostome ancestor had at least two GATA factors with distinct roles in early germ layer development: an endomesodermal GATA456 gene and an ectodermal GATA123 gene [2]. In this analysis, at least one representative was identified from each class in multiple protostome genomes, and the germ layer specific expression for each class was documented in a basal lophotrochozoan, the polychaete annelid *Platynereis dumerilii*. However, orthologous relationships for the more degenerate *C. elegans *and *Drosophila *GATA transcription factors remained unclear.

Here, we report an analysis of the complete complement of GATA factors from several newly available protostome genomes. We have identified GATA factors from nine diverse protostomes by directly searching databases from recently conducted whole genome sequencing efforts. We have conducted phylogenetic analyses using predicted protein sequences, conserved chromosomal gene order, and conserved intron/exon boundaries to better understand the evolution of protostome GATA factors. Our results provide evidence for protostome-specific expansions of GATA456 paralogs and enable us to infer the evolutionary relationships of even the most divergent *Drosophila *GATA factors.

## Results

### The complement of GATA transcription factors from newly sequenced protostome genomes

To further investigate the evolution of GATA transcription factors within protostomes, we obtained GATA gene sequences from nine newly sequenced and phylogenetically informative protostome genomes (see Materials and Methods). These include five arthropods [*Ixodes scapularis *(tick), *Daphnia pulex *(water flea), *Tribolium castaneum *(beetle), *Apis mellifera *(bee), and *Anopheles gambiae *(mosquito)], one nematode (*Caenorhabditis briggsae*), and three lophotrochozoan [*Lottia gigantea *(limpet), *Capitella capitata *(polychaete), *Schmidtea mediterranea *(flatworm)] genomes. For almost all of these collected GATA transcription factor genes we identified and assembled the complete dual-zinc finger domain for further analyses. We believe that these retrieved GATA genes represent the complete GATA gene complement within each analyzed genome (see Materials and Methods, Additional File 1).

Each ortholog was initially assigned to either the ancestral bilaterian GATA123 or GATA456 class (see Introduction), based upon reciprocal best hit BLAST analysis (see Methods). With the exclusion of the nematode *Caenorhabditis briggsae *(discussed below), each of the additional protostome genomes appeared to possess a single GATA123 ortholog and three GATA456 paralogs. In the four insect genomes, as well as in the single annelid (*Capitella capitata)*, a fourth highly divergent GATA456 paralog was detected. Thus, our initial genome wide search indicated the existence of one single copy GATA123 gene and multiple copies of GATA456 genes within these additional protostome species.

### Molecular phylogenetic analysis defines multiple distinct GATA456 clades within arthropods

Resolving the phylogenetic relationships of GATA transcription factors present in *Drosophila *has been difficult due to their highly divergent gene sequences. Previous work had shown that *Drosophila *possesses an obvious GATA123 ortholog, *grain*, as well as an unambiguous GATA456 ortholog, *pannier *[3]. However, the placement of the three remaining *Drosophila *GATAs (*serpent*, *GATAd*, and *GATAe) *has been uncertain due to extensive sequence divergence within the generally well-conserved dual zinc finger domain. The *Drosophila *GATA genes *serpent *and *GATAe *have been proposed to be derived GATA456 orthologs due to their roles in endoderm and/or mesoderm development [2]. However, like some vertebrate GATA123 genes, *serpent *also has roles in blood development suggesting that *serpent *may be orthologous to all vertebrate GATA genes [4,5].

**Figure 1:**
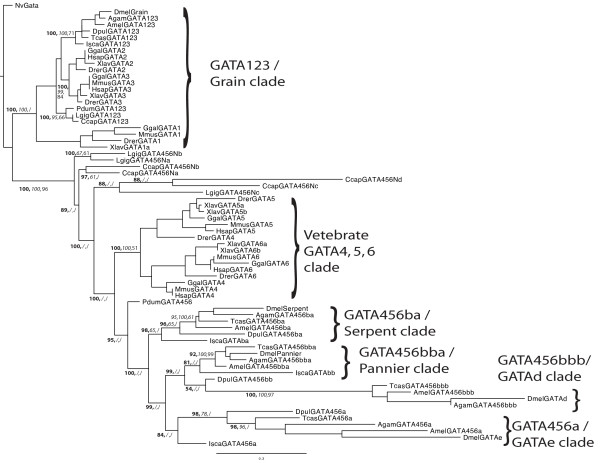
**Phylogenetic analysis of GATA Transcription Factors**. Gene phylogeny based on a combined molecular phylogenetic analysis using Maximum Likelihood (ML), Bayesian Analysis (MB), and Neighbour Joining (NJ) methods. Genes are prefixed by a short abbreviation for the organism (1 letter for genus, three for species). Topology and branch lengths were generated using the PhyML-aLRT program, and branch support for key nodes is shown in bold (ML, SH-like aLRT statistic), italics (MB, posterior probabilities), and plain text (NJ, bootstrap percentiles). Inferred arthropod and vertebrate clades are marked by brackets to the right.

We now can resolve the uncertain *Drosophila *GATA factor relationships by including sequences from additional arthropod genomes. The phylogenetic tree in Figure 1 represents the combined results of maximum likelihood (ML), Bayesian inference (MB), and distance based (NJ) analyses (see Materials and Methods). This tree was rooted with the sole GATA transcription factor in the cnidarian *Nematostella vectensis *(NvGATA), which appears to be equally related to GATA123 and GATA456 genes [2]. This analysis confirms the existence of two separate branches of bilaterian (protostome and deuterostome) GATA factors, a clade of GATA123 genes and a clade of GATA456 genes. It replicates previous results [2], but now with substantially increased support due to the presence of additional and more conserved GATA sequences. With the addition of multiple arthropod sequences, the diverged *Drosophila serpent, GATAe*, and *GATAd *now unambiguously group within the larger GATA456 clade. The GATA123 versus GATA456 groupings are well supported by both ML and MB analyses, though not by NJ analysis, which shows lower bootstrap support due to the occasional grouping of the NvGATA within the GATA123 clade. We presume this is a possible short-branch attraction artifact, due to the relatively low sequence divergence for the GATA123 and NvGATA genes. However, consistent with our results from the reciprocal best hit BLAST analysis (see above), all of the arthropod genomes encode sole GATA123/Grain like orthologs but multiple GATA456 like paralogs.

Our more inclusive phylogenetic analysis also reveals orthologous groups among the arthropod GATA456 paralogs. The four *Drosophila *GATA456-like transcription factors are co-orthologs of the vertebrate GATA456 family, and appear to have independently expanded early in arthropod evolution. The *Drosophila *GATA456 paralogs are members of four distinct clades, each containing a single paralog from every analyzed insect genome. Furthermore, three of these insect GATA456 paralog groups appear to be conserved within the arthropods, as one paralog for each of the three clades are found in the crustacean *Daphnia*, and in the chelicerate/tick *Ixodes*. We have named the three common arthropod clades as GATA456a/GATAe, GATA456ba/serpent, and GATA456bb, using a nomenclature discussed below (see Material and Methods). GATA456bb appears to have undergone an insect-specific duplication (see Discussion), resulting in the GATA456bba/pannier and GATA456bbb/GATAd clades in insects, and hence explaining the fourth insect GATA456 paralog.

While our results support four distinct clades within the arthropod GATA456 subfamily, the deeper evolutionary relationships among these clades remain uncertain. NJ and MB analyses represent the internal relationships between the four GATA456 paralogous clades as an unresolved basal polytomy, despite the resolution of four external GATA456 clades. However, the ML analysis suggests additional interclade relationships, as shown in the tree in Figure 1. All of the arthropod GATA456 paralogs appear to form a distinct and well supported clade. The insect specific GATA456bbb clade groups closely with GATA456bba clade, and therefore appear to be duplicates from a common GATA456bb gene. This topology also suggests that theGATA456bb factors appear more closely related to GATA456a orthologs than to the GATA456ba ortholog group, but we have found no additional evidence to support this relationship.

### An arthropod GATA456 paralog cluster: Synteny reveals orthologous relationships

**Figure 2:**
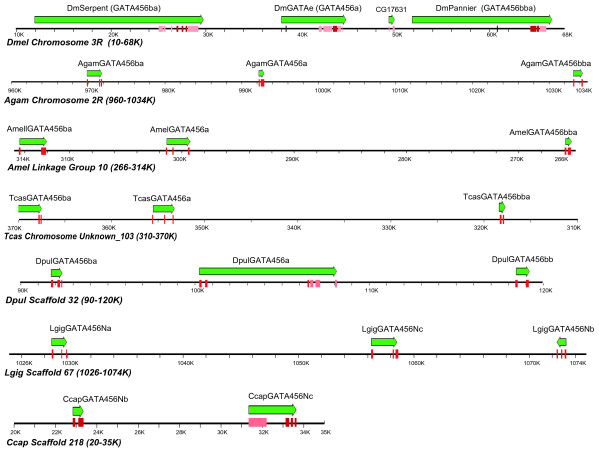
**Synteny of GATA456 paralogs in arthropods and lophotrochozoans**. Linked GATA456 orthologs are shown within genomic regions ranging from 30 kB to 75 kb in length. Green arrows indicate the transcribed GATA456 gene regions and transcriptional direction. The full extent of the transcribed region is known only for well-characterized genomes like *Drosophila*. Predicted coding sequences (CDS) are shown in pink; the conserved dual-zinc finger domain in red.

To better understand the evolutionary relationships of arthropod GATA456 paralogs, we examined the syntenic relationships among the different arthropod GATA factors and discovered a conserved linkage of GATA456 paralogs. As shown in Figure 2, we found that three of the *Drosophila *genes – *serpent, GATAe*, and *pannier *– are clustered within a 47 KB region on the *Drosophila *2R chromosome. We have identified a similar cluster of three tightly linked GATA456 paralogs in other arthropod genomes, including additional insects and a crustacean (see Figure 2). Gene orientation within the cluster is fully conserved, and the gene order follows the predicted orthology suggested by the clades in our molecular phylogenetic analysis (see above). As this cluster is conserved in all analyzed insects and a crustacean, we infer that this cluster arose at least as early as the pancrustacean ancestor, some 420 million years ago. No additional syntenic relationships were found when comparing the nearest upstream and downstream genes from each of the five assembled arthropod genomes, suggesting that gene order within this GATA456 paralog cluster is more conserved than within surrounding chromosomal regions.

The conserved linkage suggests an origin from two tandem duplication events of a single ancestral GATA456 transcription factor. This cluster includes an unambiguous GATA456 ortholog (*pannier*) [2,3], further supporting our phylogenetic inference that these three homologs are all GATA456 paralogs. Furthermore, the weak homology of the three identified tick GATA transcription factors to each of these paralogs suggests that this three-gene cluster may have existed in the last common arthropod ancestor. However, our initial attempts at local contig assembly (see Materials and Methods) have failed to find linkage for the tick GATA genes. GATA gene linkage in the tick should soon be resolved, pending assembly of the whole tick genome.

### A unique intron/exon structure for each of the three arthropod GATA456 clades

**Figure 3:**
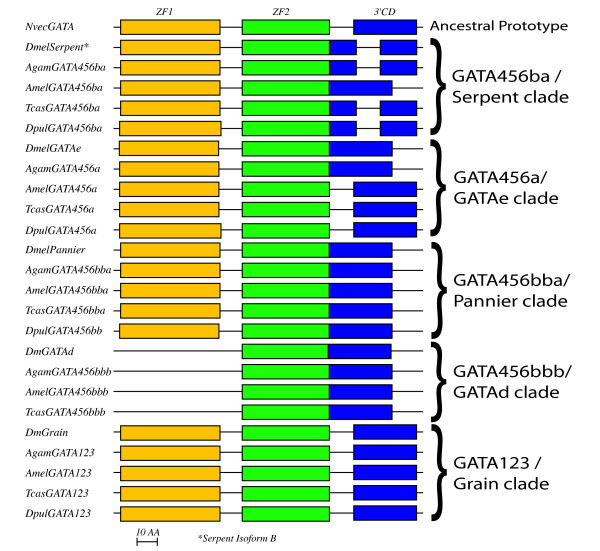
**Intron/exon Structure of arthropod GATA conserved domains**. Schematics of the exon structure for the conserved dual-zinc finger domain of the arthropods GATA transcription factors. The ancestral prototype for this conserved domain consists of three exons with well-conserved boundaries, represented here by the sole *Nematostella vectensis NvecGATA*. The first exon (ZF1 in yellow) consists of the first DNA binding zinc-finger, whereas the second (ZF2 in green) contains the second DNA binding zinc-finger domain. The third exon (3'CD in blue) contains the GATA N-terminal activation domain described for some species.

The ~135 AA dual-zinc finger domain that defines the broader GATA transcription factor gene family, including both GATA123 and GATA456 homologs [3], is encoded by three exons with similar intron/exon boundaries that are found in the sole cnidarian GATA gene, and in all deuterostome GATA genes we have examined (data not shown). We infer that the ancestral GATA transcription factor gene contained these three exons (see Figure 3, Additional File 1). An N-terminal exon (ZF1, ~50 AA) encodes the first zinc-finger, and a middle exon (ZF2, ~54AA) encodes the second DNA binding zinc-finger domain. The C-terminal exon (3'CD) encodes a conserved stretch of ~30 AA, after which conservation sharply drops.

Although the exon structure of this dual zinc finger domain is well conserved in most GATA homologs, many GATA genes in the fruitfly *Drosophila melanogaster *appear to lack the first zinc finger. In *Drosophila*, the first *GATAe *zinc finger is highly divergent, and *GATAd *lacks any sign of the first zinc finger. *Serpent *was initially thought to be a single zinc-fingered protein [5] – two of the three splice variants for *serpent *lack the first zinc finger – but a more complete analysis identified the *serpent *isoform B with a complete conserved domain [6]. A lack of a first zinc finger in many GATA factors within *Drosophila*, as well as *C. elegans *(see below), could suggest that these GATA factors evolved from an ancestral sequence encoded by a single zinc finger [3,5]. However, our examination of additional arthropod GATAs indicates that independent losses of the first zinc-finger have occurred. Both zinc-finger exons appear intact for the three ancestral arthropod GATA456 genes in the additional arthropod species. Only the insect specific GATA456bbb/*GATAd *orthologs consistently lack the first zinc finger exon; however, our analysis shows these are relatively recent duplicates, and hence must have arisen from dual-zinc fingered GATA456 factors. Therefore, we conclude that the absence of the first exon and zinc finger in some *Drosophila *GATA genes is a derived rather than an ancestral trait.

Our analysis of GATA gene structure also suggests a derived loss or modification of the second (2ZF) and third (3'CD) exon boundary in many of the arthropod GATA homologs (Figure 3). 16 of the 24 identified insect and crustacean (pancrustacean) GATA homologs have undergone a modification of this exon boundary compared to the inferred ancestral sequence. The second and third exons in all of the GATA456bbb/GATAd and GATA456bba/pannier orthologs are fused. The GATA456a/GATAe genes have retained the ancestral state for the intron/exon domains, though they have also fused their second and third exons in dipteran insects. The GATA456ba/serpent genes (except the honey bee *Apis) *also contain a second intron in the conserved domain, yet the boundaries of this second intron do not appear conserved.

The unusual intron/exon structure of the GATA456ba/serpent genes suggests they may have resulted from an initial fusion of the second and third exon, and the subsequent introduction of a new intron. We have identified four examples of insect and *Daphnia *GATA456ba/serpent homologs in which the first 13 AA from the third exon (3'CD) are now encoded within the middle exon (2ZF). The high degree of sequence conservation implies a transfer between the original coding exons, as opposed to a loss of the beginning of the third exon and gain of surrounding genomic sequence at the end of the middle exon. This presumably rare sequence of events likely occurred only once, and was then preserved within GATA456ba/serpent orthologs. The one exception, *AmelGATA456ba*, likely represents an additional intron loss.

To summarize, the clades of arthropod GATA456 homologs defined by molecular phylogeny also exhibit clade-specific intron/exon structures. This correspondence provides a third line of evidence in support of our proposed orthologous relationships for these genes, as suggested by both molecular phylogenetic analysis and conserved syntenic gene order. The intron/exon structure also allows us to generate deeper inferences regarding interclade relationships (see Discussion).

### Extensive gene duplication and sequence divergence within the nematode GATA family

We also analyzed the GATA genes of two nematode species, *Caenorhabditis briggsae *and *Caenorhabditis elegans*. The GATA gene family has undergone extensive duplication in nematodes, with eleven GATA factors identified in *C. elegans*, and thirteen in *C. briggsae*. These sequences display significant sequence divergence, and only the *elt-1*/GATA123 orthologs contain complete dual-zinc finger domains. The other predicted nematode GATA factors all lack the first zinc finger, similar to some of the insect GATAs.

Although the nematode GATA factors are highly derived in sequence, they resemble the arthropod GATA complement in displaying a biased expansion of the GATA456 paralogs. In a previous analysis, we assigned the *C. elegans *GATA factors to one of the two classes based upon their reported germ layer-specific function or expression. Four orthologs have roles in ectoderm (epidermis and nervous system) specification, while seven *(C. elegans*) or nine *(C. briggsae) *have roles in endomesoderm (intestine and muscle) specification (see Additional File 1). However, our phylogenetic tree (see Additional File 2) suggests that *elt-1 *is the sole GATA123 ortholog in both nematode species, and that all the remaining nematode GATAs group within the GATA456 class. The long branches of these additional GATAs, and the short regions of conserved sequence, make these inferences highly speculative. Nevertheless, our data suggests that, like the arthropods we have analyzed, both nematodes have undergone a greater expansion of GATA456 paralogs, relative to GATA123 paralogs.

**Figure 4:**
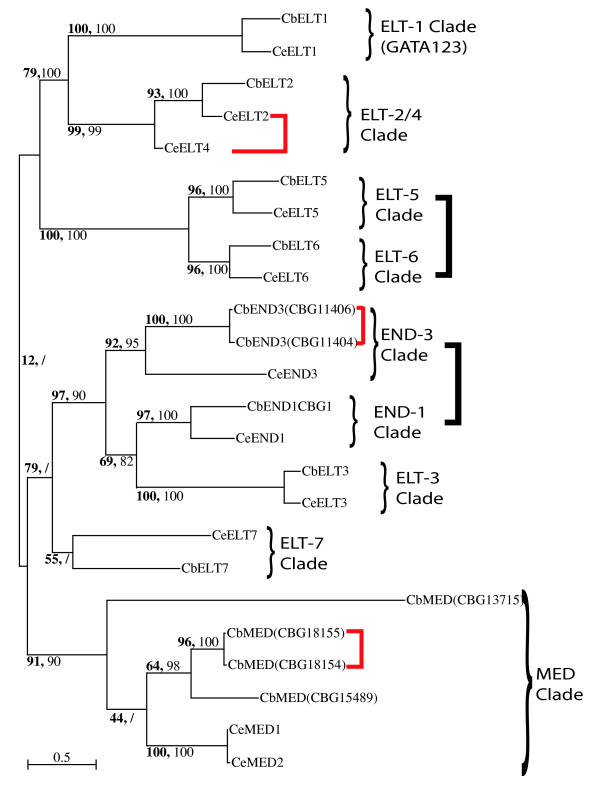
**Molecular phylogeny of nematode GATA factors**. Maximum Likelihood tree of *C. briggsae *(Cb) and *C. elegans *(Ce) GATA factors, showing both the PhyML-aLRT Chi2-based parametric statistic (bold) and Neighbor Joining Bootstrap percentiles (regular). Brackets to the right indicate the inferred ancestral clades. Black box brackets indicate genomic linkage found for both nematode orthologs in two clades, while red box brackets indicate linkage found only within one nematode species.

To evaluate the relationships between the GATA factors in the two nematode genomes, we have conducted additional phylogenetic analyses using the complete gene sequences from these two *Caenorhabditis *genomes (see Figure 4). This analysis provides clear support for nine common clades of *C. elegans *and *C. briggsae *GATAs, based upon the ability to define orthology between individual *C. elegans *and *C. briggsae *GATAs. This suggests that the last common ancestor of these two nematodes possessed at least nine distinct GATA genes. Furthermore, several nematode GATA factors appear to have resulted from more recent duplications within these clades, suggesting their duplication after the divergence of *C. elegans *and *C. briggsae *lineages some 80–110 million years ago [7]. These include *C. elegans elt-4*, *C. briggsae end-3*, and the *med *genes in both species.

We also observed chromosomal linkage of some nematode GATA genes. Most of the linked genes are the same ones identified as more recent duplicates within single clades that are specific to only one nematode species: the *C. elegans elt-4 *and *elt-2 *genes, as well as the *C. briggsae end-3 *and two of the *med *genes. Some orthologs are linked in both nematode species, including the *elt-5/6 *and *end-1/3 *orthologs, indicating a linkage retained from an ancestral *Rhabditis *species. However, these linked genes are from closely related clades, and possess long internal branch lengths suggestive of an evolutionary origin within nematodes. We conclude that the linked nematode GATAs originated from more recent nematode and *Rhabditis *specific duplication events, and do not reflect any retention of deeper ancestral gene duplicates.

### Similar GATA family gene number and linkage, but different intron/exon structures, in lophotrochozoans and arthropods

The lophotrochozoan GATA complement is similar in copy number to the arthropod GATA complement. For example, all of the analyzed lophotrochozoans possessed three or four GATA456 homologs, and a single GATA123 homolog (see Figure 1). In the flatworm, *Schmidtea mediterranea*, and the limpet, *Lottia gigantea*, we have found three GATA456 paralogs in a reciprocal best hit BLAST analysis. The annelid polychaete, *Capitella capitata*, appears to possess an additional GATA456 homolog. The annelid leech *Helobdella robusta*, has 13 predicted GATA homologs; however, the number and extreme divergence of these leech GATAs relative to other lophotrochozoan genomes appear to make these uninformative in reconstructing the ancestral annelid condition.

As in arthropods, we also identified syntenic relationships of GATA456 genes in two lophotrochozoan genomes (see Figure 2). In *Lottia*, all three of the GATA456 orthologs are contained within a 45-kilobase region, although the third gene appears to be inverted in orientation compared to the arthropod genes. In *Capitella*, two of the four GATA456 genes appear in an 11-kilobase region, though linkage to a third gene has not been found. None of the GATA genes appear linked in *Schmidtea*, consistent with the relatively degenerate nature of the flatworm GATA genes (data not shown), or perhaps a consequence of the short lengths of the currently available assembled genomic regions.

Although similar in number and linkage, it is unclear to what degree the lophotrochozoan and arthropod GATA456 duplicates can be considered orthologous. No relationships between individual arthropod and lophotrochozoan GATA456 duplicates are apparent from our molecular phylogenetic analyses.

It is also unclear to what degree these lophotrochozoan GATAs are related to one another. The *GATA456Nc *genes appear to be orthologous across *Capitella *and *Lottia *in both ML and MB analysis, and the *CcapGATA456Nd *is supported as a more recent duplicate of the *CcapGATA456Nc *gene in the ML analysis. However, the two well-conserved GATA456 duplicates from both *Capitella *and *Lottia *(*Na, Nb) *group more closely within each species then across species, suggesting these could be recent lineage-specific duplicates. The sole *GATA456 *identified in another polychaete, the *Platynereis dumerilii PdGATA456*, does not branch near the other lophotrochozoan GATAs, instead branching as the closest outgroup to the arthropod GATA456 clade. Finally, when we examined the intron/exon structure of all of the analyzed annelid (*Capitella capitata*), mollusk (*Lottia gigantea*), and flatworm (*Schmidtea mediterranea) *GATA genes, we found little evidence for the extensive modifications seen for the arthropod GATA456 homologs (see Additional File 1). Thus, while the orthologous relationships between arthropod GATA456 factors appear well supported, additional information will be needed to resolve the evolutionary relationships of lophotrochozoan GATA456 factors.

## Discussion

We have identified the complete complement of GATA factors from nine additional protostome genomes, and we have reconstructed the evolution of protostome GATA factors using multiple approaches. Our initial estimates of orthology, from reciprocal best hit BLAST analysis, revealed an expansion of the GATA456 paralogs in protostomes, while the GATA123 genes appear to be retained as a single copy. The inclusion of additional arthropod genomes has allowed us to confidently assign the more divergent *Drosophila *GATA factors as GATA456 paralogs. Furthermore, we have demonstrated widespread linkage of many GATA456 duplicates, suggesting a mechanism of gene duplication via tandem duplication events and providing further evidence in support of duplicate-orthology. Finally, we infer from changes in intron/exon structure the sequence of gene duplications that produced the GATA456 paralogs in arthropods.

### Overview of the evolution of the protostome GATAs

**Figure 5:**
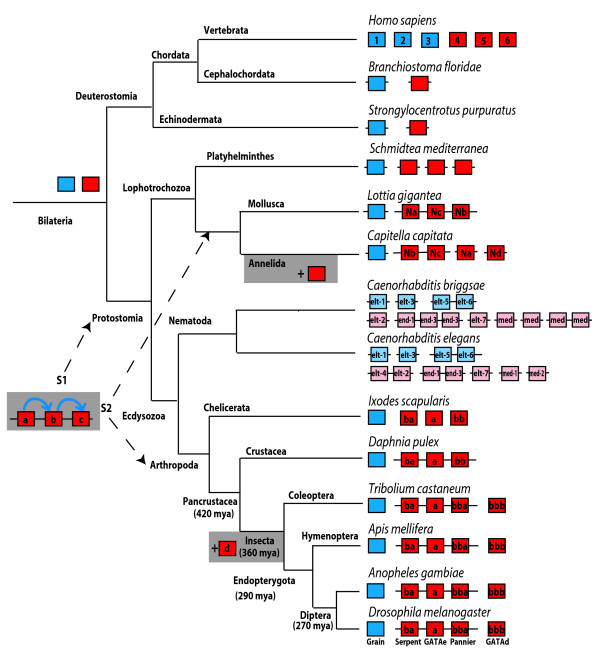
**Evolution of protostome GATA factors**. Alternate scenarios for the early/orthologous (S1) or late/convergent (S2) evolution of the arthropod and lophotrochozoan GATA456 gene clusters. Names at a node represent the name of the clade of organisms, and the time since the last common ancestor is given for some nodes in million years (mya) (tree topology, dates, and nomenclature from [19–21]). Boxes represent individual GATA genes, with GATA123 orthologs in blue, and GATA456 orthologs in red. Light shaded nematode GATAs represent predictions based mainly upon functional conservation. Linked genes are represented with a connected line, and identified via their terminal letters (see Materials and Methods).

We suggest two alternative scenarios for the evolution of the GATA transcription factors in protostomes (Figure 5). In the first scenario, the GATA456 family expanded very early during protostome evolution. In support of this scenario, there are similar numbers of GATA456 paralogs in lophotrochozoans (three, with a fourth in *Capitella*) and arthropods (three, with a fourth paralog in insects), even though they each appear to possess single GATA123 orthologs. Furthermore, the chromosomal linkage of GATA456 paralogs, not only in arthropods but also in the mollusk, *Lottia*, and partially in the annelid, *Capitella*, is suggestive of a deep origin.

However, our analyses more strongly support a second scenario, in which there have been independent duplications of a single GATA456 ortholog in both arthropods and lophotrochozoans. This second scenario is suggested by the lack of affinities between individual lophotrochozoan and arthropod GATA456 paralogs in molecular phylogenetic analyses. One possibility is that this cluster arose very close to the lophotrochozoan-ecdysozoan split, allowing little time for sequence divergence and retention of phylogenetic information between the GATA456 paralogs. Nevertheless, the conserved modifications of intron/exon boundaries between orthologous arthropod GATA genes suggest that intron loss and gain occurred before the duplication of certain arthropod GATA456 paralogs (see below). Because all but one of the analyzed lophotrochozoan GATA456 genes have retained the ancestral intron/exon structure, we conclude that either the GATA456 paralog clusters in lophotrochozoans and arthropods have independent origins, or that GATA456 genes in arthropods have undergone repeated and convergent intron losses.

### Orthology and evolutionary birth-order of the arthropod GATA gene complement

We have used multiple lines of evidence to determine the origin and relationships of the more degenerate *Drosophila *GATA factors. A reciprocal best hit BLAST analysis suggested that only one (*Grain*) of the five *Drosophila *GATAs belongs to the GATA123 class, while the remaining four are in the GATA456 class. Additionally, in multiple phylogenetic analyses all five *Drosophila *GATAs formed single clades with other arthropod homologs, suggesting orthologous relationships between the GATA genes within each clade. Orthologous genes for each of three fly GATA456 genes – *Serpent, GATAe*, and *Pannier – *are present throughout arthropods. However, the fourth *GATA456 *paralog, *GATAd*, appears to be an insect-specific duplicate found only in the beetle, bee, mosquito, and fruitfly genomes.

Additional evidence for the orthology of the four arthropod GATA456 genes comes from the observed conservation of gene order within a GATA456 paralog cluster among arthropods. The *Drosophila *GATA genes *Serpent, GATAe*, and *Pannier *are present within a tightly linked cluster, as are their best-hit orthologs in all the arthropod genomes we analyzed. Moreover, the relative orientations of these three genes are the same in every analyzed arthropod genome. In contrast, the insect specific GATA456 gene, orthologous to *GATAd*, is not linked in any of the four analyzed insect genomes. Thus, the gene order is consistent with the predicted orthology defined by our phylogenetic analysis.

A third independent line of evidence for the orthologous relationships among arthropod GATAs emerged from our comparative analysis of the genomic intron/exon structures. The ancestral condition for the genomic structure of the conserved dual zinc finger domain of GATA transcription factors is three exons with conserved intron/exon boundaries, as found in all vertebrate, lophotrochozoan, and cnidarian GATAs analyzed. In contrast, arthropod GATA456 genes exhibit extensive modifications from this ancestral genomic organization. However, the suspected orthologs among the arthropod GATA456 homologs are united by unique clade specific intron/exon structures.

**Figure 6:**
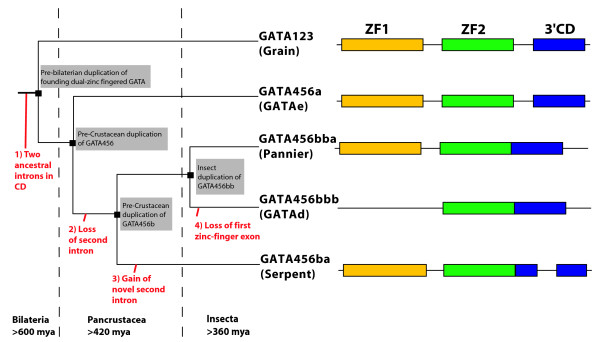
**Intron/exon structures define relationships and evolutionary birth order of arthropod GATA paralogs**. The intron/exon structures for the arthropod GATA456 genes suggest a birth order (as described in the discussion). The dotted line represents emergence of the last common bilaterian, pancrustacean, and insect ancestors. The red text represents points of intron/exon modifications, while the grey box describes the timing of gene duplication events. Intron/exon structures represent the inferred state for each paralog in the last common insect ancestor.

The observed synteny, as well as the pattern of intron losses and gains of the arthropod GATA factors, also provide an explicit mechanism for gene expansion via tandem-duplication and suggest an evolutionary birth order for the GATA genes during the expansion from one ancestral to four GATA456 homologs in arthropods. As illustrated in Figure 6, we can infer the following sequence of duplication events. GATA456a/GATAe and the GATA456b precursor would have first arisen from an initial tandem duplication of a GATA456 gene that possessed the ancestral intron/exon structure. GATA456b then lost the second intron, resulting in a secondary state. The subsequent duplication of GATA456b formed GATA456ba/Serpent and GATA456bb. Gain of a novel second intron by GATA456ba/Serpent produced a third state. Following the divergence of insects from a pancrustacean (insect and crustacean) ancestor, a duplication of GATA456bb generated both the GATA456bba/Pannier GATA456bbb/DmGATAd genes. GATA456bba genes appear to retain the second state, but the GATA456bbb genes appear to have lost the first exon (ZF1) early.

Our analysis shows the utility of such rare genomic changes as additional characters for resolving the relationship between deep branches in gene phylogenies. In this case, combining synteny and gene structure analysis with molecular phylogeny helped to resolve not only the obscure phylogenetic relationships of the highly derived *Drosophila *GATA456 genes, but also suggests the sequence of gene duplications that produced this gene family in arthropods. This evolutionary scenario makes predictions about the sequences, intron/exon structure, and syntenies of GATA456 genes within arthropod phylogeny that can now be further tested by obtaining genome sequences from additional arthropods. This scenario also predicts similarities in expression and function of the orthologous GATA genes in arthropod development.

The identification of clear arthropod orthologs to *Drosophila *GATA factors also allows us to infer the origin of metazoan single-zinc fingered GATAs. Non-metazoan GATA factors, such as the fungal AreA proteins, generally possess single zinc-fingers, but most metazoan GATA factors possess dual zinc fingers. However, some invertebrate GATAs (e.g. *Drosophila GATAd *or *Serpent *isoforms) possess only single zinc fingers that might indicate their independent origin from single-zinc fingered ancestors. However, as described above, other arthropods possess orthologs of single finger *Drosophila *genes with two zinc fingers, indicating these genes all arose from dual-zinc fingered ancestral sequences. This conclusion is further supported by molecular phylogenetic analyses suggesting that all metazoan GATA factors are equally related to fungal outgroups (W.J.G, unpublished results). Finally, we also have been able to identify highly conserved individual amino acids that are diagnostic for individual arthropod orthologs and may be useful for identifying orthologous GATA factors in partially sequenced arthropods genomes (see Additional File 3).

### Unresolved GATA456 orthology in other protostome phyla

While we can use both gene sequence and synteny to infer GATA456 factor phylogeny among arthropods, it remains unclear whether the GATA456 expansion in arthropods is related to, or independent of those observed in other protostomes. For example, we can define nine *Caenorhabditis *clades, but we have little understanding of how these relate to the four predicted ancestral arthropod GATA genes. Several of the nematode GATA genes are tightly linked, but most of these appear to involve very recent duplications. While sequenced genomes are now becoming available for additional nematode species, currently the data from the well supported *C. elegans *and *C. briggsae *GATA sequences provide the best platform to launch future inquiries into GATA gene family evolution in nematodes.

Likewise, we cannot fully resolve how lophotrochozoan GATA456 paralogs are related to each other, or to *Drosophila *GATA456 paralogs, despite similar copy-number and linkage. In each of three lophotrochozoan genomes, we identified three or more GATA456 paralogs. Two and three of these GATA456 paralogs are linked in an annelid (*Capitella) *and molluskan (*Lottia) *genome, respectively. In our molecular phylogenetic analysis, the *CcapGATANc *and *LgigGATANc *form a clade, and are both part of the GATA456 clusters in either organisms, suggesting these may represent orthologs across molluscs and annelids. However, our molecular phylogenetic analyses fail to define additional relationships for the GATA456 paralogs in lophotrochozoans, or between lophotrochozoans and arthropods.

Additionally, the sequence and expression of four of the five *Capitella *GATA factors was recently characterized [8]. They name these factors *CapI-gataA, CapI-gataB1, CapI-gataB2*, and *CapI-gataB3*, which appear to correspond to *CcapGATA123*, *CcapGATA456Na*, *CcapGATA456Nc*, and *CcapGATA456Nb*, respectively. Interestingly, the mRNA expression of the GATA123 ortholog was reported to be restricted to ectodermal derivatives, while the expression of the three GATA456 paralogs was described within nested mesendodermal territories. These results provide additional evidence for a class-specific germ layer expression and expansion of GATA456 versus GATA123 transcription factors [2].

One path to resolving gene-family relationships in other protostome phyla is to survey additional taxa, with a focus on slow evolving genomes and appropriate phylogenetic position. A better understanding of the *Drosophila *GATA factor evolution was possible only after additional arthropod genomes from suitable phylogenetic branches were included, many of which possessed less derived GATA sequences. However, the arthropods currently are an exceptionally well-sampled protostome phylum, and our findings suggest that the current sampling of lophotrochozoan genomes is not sufficient to resolve their gene family evolution. In order to extend our findings in arthropods to other phyla, it will be important to survey additional protostome genomes. Several ecdysozoan genomes have been targeted for whole genome sequencing, including those from basal taxa such as tardigrades and priapulids. Genome sequences from additional nematode species may help resolve the current ambiguity about the relationships of the *Caenorhabditis *GATAs.

## Conclusion

In this study, we identified and examined the complete complement of GATA factors from nine newly sequenced protostome genomes. We have reconstructed the evolutionary relationships of these protostome GATAs using complementary forms of phylogenetic inference, including molecular phylogenetic analysis, genomic linkage and an examination of intron/exon boundaries. Our analysis indicates that protostome genomes have a single GATA123 ortholog, but multiple GATA456 paralogs. Furthermore, by including many arthropod genomes, we have been able to define orthology for the more degenerate *Drosophila *GATA factors, including assigning the *GATAd, GATAe*, and *serpent *genes conclusively as GATA456 co-orthologs. Our examination of intron/exon structure modifications suggests a birth order of GATA456 paralogs, which could not be resolved in molecular phylogenetic analyses. This analysis has also identified similar tightly linked clusters of three GATA456 orthologs in both arthropods and lophotrochozoans, but additional taxa sampling will be required to define gene family relationships among diverse protostome phyla.

## Methods

### Identification of the conserved domains of the GATA transcription factor complement

To identify putative GATA conserved domains, whole genome traces were downloaded to a local database and searched using two previously described *Platynereis *GATA factors and TblastN with each individual genome. Genome sequence from *T. castaneum *(Tcas_2.0) and *A. mellifera *(Amel4.0) was obtained from the Baylor College of Medicine Human Genome Sequencing Center [9]. *I. scapularis *(iscapularis.TRACE-WIKEL.june07) and *A. gambiae (*AgamP3) genome sequence was obtained from the VectorBase [10]. *D. pulex, C. capitata*, and *L. gigantea *sequence data (v.1.0) was obtained from the US Department of Energy Joint Genome Institute [11]. *S. mediterranea (v.3.1) *sequence data was produced by the Genome Sequencing Center at Washington University School of Medicine in St. Louis [12].

The TblastN hits from the genomic trace archives were validated and grouped using subsequent blast analyses. First, TblastN hits were validated by blastx against the Genbank NR genome, with a positive hit showing highest similarity to GATA sequences in other organisms. Validated hits were then clustered, using blastn to search for like hits in the organism's trace archive, using these to group all positive traces and remove duplicates from the list of positive TblastN hits. The best deuterostome TblastN hit from each of the blastx analyses was recorded, and used for reciprocal best hit BLAST analysis to assign the initial orthology to known deuterostome classes. This process was repeated until no additional exons could be identified.

To assemble the individual exons, we used two distinct methods. In cases where a genome assembly was publicly available, contigs containing these exons were identified by blastn and compared to define the assembled exon structure for individual genes. In cases where no genome assembly was available, we attempted to first connect these exons by searching for traces with overlap between two exons. In the case where no single trace could be identified to connect two exons, we performed chromosome walks on the individual exon using the Tracembler program [13]. These larger contigs, which was based upon overlapping sequence and also mate-pair relationships, were then used to determine linkage between genes.

### Nomenclature for additional protostome GATA factors

We have named these additional protostome GATA factors using a nomenclature that reflects our inferred evolutionary relationships. In some cases, such as for the arthropod and insect GATAs, we can infer not only orthologous relationships, but also the sequence of duplications that lead to additional paralogs. In these cases, we use a binary naming system to describe each gene speciation event, adding an 'a' to one duplicate, and 'b' to the other. In cases where only uncertain orthology is inferred, we describe the orthology following our convention, and describe multiple, uncertain orthologs using a capital 'N', followed by letters ranked by their degree of sequence conservation (from most conserved to least conserved).

### Molecular phylogenetic analysis

A sequence file was made for each of the GATA genes using the conserved dual-zinc finger domain, consisting of the two zinc finger exons and the N-terminal portion of the following exon. These sequences were aligned using Clustalw [14], and then manual improvements were made in MacVector (see Additional File 4). Maximum likelihood analysis was conducted using PhyML-aLRT [15,16] using a JTT model of evolution, and branch support given by the aLRT CHI2-based parametric statistic. Bayesian Inference was conducted using the MrBayes v3.1 [17], using the JTT model of evolution. The results are a consensus of two-converged runs of 3,000,000 generations, and branch supports given as posterior probabilities. Neighbor joining distance-based analyses was conducted using the MacVector program (v7.2.3) [18], and the support given by bootstrap percentiles of 10000 replicates. For the nematode GATA factors, the complete sequence for each factor was aligned using Clustalx, a tree was generated using PhyML-aLRT, and includes support from both the PhyML-alrt CHI2-based parametric statistic and Bootstrap percentiles from a Neighbor Joining analysis in MacVector.

## Authors' contributions

WG performed sequence and evolutionary analyses. WG and SQS designed the study and analyzed the data. BB and SQS conceived and supervised the study. WG, BB, and SQS drafted the manuscript. All the authors read and approved the final manuscript.

## Supplementary Material

Additional file 1 Protostome GATA gene list. This file contains the annotated list of GATA Transcription factor sequences identified and analyzed in this paper. Click here for file

Additional file 2 ML analysis of conserved GATA dual-zinc finger domains of protostomes, including nematodes. This tree represents a Maximum Likelihood phylogenetic analysis of the conserved GATA dual-zinc finger domains including nematodes. Click here for file

Additional file 3 Signature Amino Acids for GATA orthologs. This file contains an annotated alignment of the consensus sequences for GATA conserved domains from various orthology groups. Click here for file

Additional file 4 Alignment of conserved GATA dual-zinc finger domains. The FASTA formatted alignment of the conserved GATA dual-zinc finger domains. Click here for file
