# Supplementary material for: The evolution of protostome GATA factors: Molecular phylogenetics, synteny, and intron/exon structure reveal orthologous relationships
Source: BMC Evol Biol. 2008 Apr 15;8:112. doi: 10.1186/1471-2148-8-112 (PMC2383905; doi:10.1186/1471-2148-8-112)
Supplement: Additional file 1 — Protostome GATA gene list. This file contains the annotated list of GATA Transcription factor sequences identified and analyzed in this paper. [file 1471-2148-8-112-S1.pdf]

Additional File 1: PrGATAs GeneList

The following is a complete list of conserved dual-zinc finger domains of identified GATA factors, with the sequences shown as individual exons. The ancestral prototype for this conserved domain consists of three exons with well-conserved boundaries, as is shown below for the *Nematostella* GATA. This ancestral genomic configuration is also found in all mollusc (*Lottia gigantea),* flatworm (*Schmidtea mediterranea),* all but one of the annelid (*Capitella capitata)*, as well as all of the vertebrate GATA sequences (data not shown). The first exon (ZF1) consists of the first DNA binding zinc-finger, and the second (ZF2) contains the second DNA binding zinc-finger domain. The third exon (3’CD), which contains the GATA N-terminal activation domain described for some species, is also included. Although the overall domain structure is well conserved across most GATA homologs, the boundaries of many of the arthropod GATA456 paralogs have shifted (see Results). To show the conserved domain sequence of each of the GATA CDs we have underlined, italicized, and bolded the ZF1, ZF2, and conserved region of the 3’CD, respectively.

Nematostella vectensis **(cnidarian, sea anemone)**

Sole NvGATA at 2253K-2258K on scaffold_2 (JGI:Nemve1/Scaffold_2)

>NvecGATA
5’ Exon: (Sca2:2253649-2254285)
METVNSEHRWIHPISGQHDSPHGASSSDRMQPTNCSGGSVNIVEPTQLLPPDEVDVFFHHLDGSGNSGNWS
YAAGPRAYRPSMCQMAAHGPTAFQDPQSQASPPGSCSRVFLPTARVPGSPVCRPHFHTPIQWIESKPAPGL
HCSTPSAASVWRPPFQQSHRGNPMAASGVSSASGVSHSSSHLFSFPPTPPKETFGSMDGGPLSSDFFSYG
ZF1: (Sca2:2255140-2255290)
DGRECVNCGATSTPLWRRDGSGHYLCNACGLYHKMNGSSRPLIKPKRRL
ZF2: (Sca2:2255808-2255938)
SAARRAGTSCANCHTTQTTLWRRNQNGDPVCNACGLYWKLHA
3’CD: (Sca2:2257827-2258186)
VNRPLSMKKDGIQTRNRKVSSKSKNKNK*NVKQEPKLGDDLKLSSSSPLMNQTSVISSSIISPMGHNGIHTP*
TAFSRPSMPGIPGIHPGLYGPIIPAPTSSAGSTYNSSSTPPSSYSSTI

Tribolium castaneum **(insect, beetle)**

TcasGATA456 Cluster at 318-371K on UnknownLG 103 (CH476355.1)

>TcasGATA456ba *Drosophila Serpent ortholog*
LIFQLVDPRAEYFTEGRECVNCGAIDTPLWRRDGTGHYLCNACGLYHKMNGMNRPLVKQPRRLSASRRVGL
TCTNCHTSTTSLWRRNTVGEPVCNACGLYFKLHGVNRPLAMKKDSIQTRKRKPKGSKDSNSRNALTNALES
TINNIKLEQSLPSVKLEHSSLGWSL

ZF1: (ChrUn_103:370259-370071)
LIFQLVDPRAEYFTEGRECVNCGAIDTPLWRRDGTGHYLCNACGLYHKMNGMNRPLVKQPRRL
ZF2: (ChrUn_103:367882-367721)
SASRRVGLTCTNCHTSTTSLWRRNTVGEPVCNACGLYFKLHGV***NRPLAMKKDSI***
3’CD: (ChrUn_103:367668-367519)
QTRKRKPKGSKDSNSRNALTNALESTINNIKLEQSLPSVKLEHSSLGWSL

>TcasGATA456a *Drosophila GATAe ortholog*
KECVNCGASVTPLWRRDGTGHYLCNACGLYNKINGVNRPPVRPPKKRNGVQCANCKTGNTTLWRRNNQGEP
VCNACGLYFKLHNVNRPLSMKKEGIQTRKRRPKSSNSHSQPAPSTSQGLIPARMGEALIIHNY

ZF1: (ChrUn_103:367668-367519)
KECVNCGASVTPLWRRDGTGHYLCNACGLYNKINGVNRPPVRPPKK
ZF2: (ChrUn_103:354391-354260)
QPGPRRNGVQCANCKTGNTTLWRRNNQGEPVCNACGLYFKLHNV
3’CD: (ChrUn_103:353496-353350)
NRPLSMKKEGIQTRKRRPKSSNSHSQPAPSTSQGLIPARMGEALIIHNY*

>TcasGATA456bba *Drosophila Pannier ortholog*
GICFLVADGSMEFQFGEGRECVNCGAISTPLWRRDGTGHYLCNACGLYHKMNGMNRPLIKPSKRLVSTATR
RLGLCCTNCGTRTTTLWRRNNDGEPVCNACGLYFKLHGVNRPLAMRKDGIQTRKRK

ZF1: (ChrUn_103:318322-318122)
GICFLVADGSMEFQFGEGRECVNCGAISTPLWRRDGTGHYLCNACGLYHKMNGMNRPLIKPSKRLVS
ZF2+3’CD: (ChrUn_103:318023-317787)
*TATRRLGLCCTNCGTRTTTLWRRNNDGEPVCNACGLYFKLHG***VNRPLAMRKDGIQTRKRKPKKPVGGERDD**
SSSASVEG

Additional GATA456 on Linkage Group 8 (CM000283.1)

>TcasGATA456bbb *Drosophila GATAd ortholog*
CTNCGTQTTTIWRRNMKGEMVCNACGLYYKLHGIDRPHTMRRDTIHTRRRARRVDLSCTNCGTQTTTIWRR
NMKGEMVCNACGLYYKLHGIDRPHTMRRDTIHTRRRR

ZF2+3’CD: (Lnk8:10187054-10187227)
*ARRVDLSCTNCGTQTTTIWRRNMKGEMVCNACGLYYKLHGI***DRPHTMRRDTIHTRRRR**

Tribolium GATA123 on UnknownLG 4 (CH476256.1)

>TcasGATA123 *Drosophila Grain ortholog*
SSGQVCRSHFHAPLHPWLSDGTKPLGHSGWVPFGGGDPDKSQSPSSAPGQPHNIFSFPPTPPKDSTPDSLE
GRECVNCGATSTPLWRRDGTGHYLCNACGLYYKMNGQNRPLIKPKRRLSAARRAGTSCANCKTTTTTLWRR
NQNGEPVCNACGLYYKLHNVSVNRPLTMKKEGIQTRNRK

5'CDS (ChrUn4:909015-909218)
SSGQVCRSHFHAPLHPWLSDGTKPLGHSGWVPFGGGDPDKSQSPSSAPGQPHNIFSFPPTPPKDSTPD

ZF1: (ChrUn4:918761-918913)
SLEGRECVNCGATSTPLWRRDGTGHYLCNACGLYYKMNGQNRPLIKPKRRL
ZF2: (ChrUn4:954922–955053)
SAARRAGTSCANCKTTTTTLWRRNQNGEPVCNACGLYYKLHNVS
3'CD: (ChrUn4:967059-967112)
VNRPLTMKKEGIQTRNRKLSSKSKKKKSGGSCLSLGGMMGDMMKPLDSSKGGFGGGGFGGGMGGGHPHLNA
ALHPHHAMSHWYQHSTHPQGFVSGPPPPAPPPTASYHHHMSSLSAAGLGLASNGMVS*

Apis mellifera **(insect, honey bee)**

AmelGATA456 Cluster at 265-315K on Linkage Group 10 (NW_001252977.1)

>AmelGATA456ba *Drosophila Serpent ortholog*
>NIEAEYFTEGRECVNCGAISTPLWRRDGTGHYLCNACGLYHKMNGMNRPLVKQPRRLSASRRVGTSCSNC
QTTMTSLWRRNTLGEPVCNACGLYFKLHGVNRPHTMKKDSIQTRKRKPKGGMKSSDTPIAGNVAGVSNNST
TITAANNNNNNNNNNNNNNNNNNNNSLKLEPGTNSNGIGMSLLFYRPVFLNLFESRSQLVRSRTNSIVRI*

ZF1: (Lg10:314500-314330)
NIEAEYFTEGRECVNCGAISTPLWRRDGTGHYLCNACGLYHKMNGMNRPLVKQPRRL
ZF2+3'CD: (Lg10:312493-312029)
*SASRRVGTSCSNCQTTMTSLWRRNTLGEPVCNACGLYFKLH***GVNRPHTMKKDSIQTRKRKPKGGMKSSDTP**
IAGNVAGVSNNSTTITAANNNNNNNNNNNNNNNNNNNNSLKLEPGTNSNGIGMSLLFYRPVFLNLFESRSQ
LVRSRTNSIVRI*

>AmelGATA456a *Drosophila GATAe ortholog*
SIMTVDPKECPNCAILTNVLRRDETGNYVCQNCIYAANKINGINRSSIKFQAGVRRTGVQCANCRTSNTTL
WRRNNNGEPVCNACGLYYKLHNVSNRPLSMKKEGIQTRKRKPKNNSGISGNLAGPSGMHKTEIKSDLLGEF
SVSNLS

ZF1: (Lg10:301390-301244)
SIMTVDPKECPNCAILTNVLRRDETGNYVCQNCIYAANKINGINRSSIK
ZF2: (Lg10:300799-300662)
FQAGVRRTGVQCANCRTSNTTLWRRNNNGEPVCNACGLYYKLHNVS
3'CD: (Lg10:299414-299256)
NRPLSMKKEGIQTRKRKPKNNSGISGNLAGPSGMHKTEIKSDLLGEFSVSNLS

>AmelGATA456bba *Drosophila Pannier ortholog*
TAMDFQFGEGRECVNCGAISTPLWRRDGTGHYLCNACGLYHKMNGMNRPLIKPSKRLTATRRLGLCCTNCG
TRTTTLWRRNNEGEPVCNACGLYFKLHGVNRPLAMRKDGIQTRKRKPKKTPEPNARSSTVEHEAAAAAAVS
TASSPSTGKLGALLPSWRRAEFWAVKD

ZF1: (Lg10:265859-265689)
TAMDFQFGEGRECVNCGAISTPLWRRDGTGHYLCNACGLYHKMNGMNRPLIKPSKRL
ZF2+3'cd: (Lg10:265600-265262
*TATRRLGLCCTNCGTRTTTLWRRNNEGEPVCNACGLYFKLH***GVNRPLAMRKDGIQTRKRKPKKT**PEPNARS
STVEHEAAAAAAVSTASSPSTGKLGALLPSWRRAEFWAVKD

Additional GATA456 on Linkage Group 14 (NW_001253100.1)

>AmelGATA456bbb_*Drosophila GATAd ortholog*
AKKVDMSCTNCGTMTTTIWRRNMKGEMVCNACGLYYKLHGVNRPVTMRRDTIHTRRR

ZF2+ 3'CD: (Lg14:46225-46374)
*AKKVDMSCTNCGTMTTTIWRRNMKGEMVCNACGLYYKLHGV***NRPVTMRRDTIHTRRR**

Apis GATA123 on Linkage Group 8 (NW_001253521.1)

>AmelGATA123 *Drosophila Grain ortholog*
VKQREEGRECVNCGATSTPLWRRDGTGHYLCNACGLYYKMNGQNRPLIKPKRRLSLMSLQSAARRAGTSCA
NCKTATTTLWRRNQAGEPVCNACGLYYKLHNVNRPLTMKKEGIQTRNRKLSSKSKKKKAGG

ZF1: (Lg8:233416-233270)
EGRECVNCGATSTPLWRRDGTGHYLCNACGLYYKMNGQNRPLIKPKRRL
ZF2: (Lg8:198194-198063)
SAARRAGTSCANCKTATTTLWRRNQAGEPVCNACGLYYKLHNV
3’CD: (Lg8:195670-185481)
NRPLTMKKEGIQTRNRKLSSKSKKKKAGG

<u>Anopheles gambiae</u> **(insect, mosquito)**

<u>Anopheles GATA456 cluster at 969K-104K of Chromosome 2R (NW_045682.1)</u>

>AgamGATA456ba *Drosophila Serpent ortholog*
LFTEGRECVNCGAIQTPLWRRDGTGHYLCNACGLYHKMNGMNRPLVKQPRRLVKDPVNQSFNYPHQSSARR
VGLQCSNCNTTNTSLWRRNQVGEPVCNACGLYYKLHNVNRPLAMKINFPLQSRKRKPKGSKNSDGNGKSNA
SNASANRQTNSSSSSLAETPKKT

ZF1: (Chr2R:969823-970002)
<u>LFTEGRECVNCGAIQTPLWRRDGTGHYLCNACGLYHKMNGMNRPLVKQPRRLVKDPVNQS</u>
ZF2: (Chr2R:971431-971610)
*FNYPHQSSARRVGLQCSNCNTTNTSLWRRNQVGEPVCNACGLYYKLH***NVNRPLAMKKDNI**
3'CD: (Chr2R:971685-971825)
QSRKRKPKGSKNSDGNGKSNASNASANRQTNSSSSSLAETPKKTDGK

>AgamGATA456a *Drosophila GATAe ortholog*
AAPPEHRECVNCGSSDTPLWRRDIVGHTLCNACALYTRQNPGTNRPPNRSQKAKQTVVSGTPPAQGNRRSG
VTCANCQTTTTTLWRRNNQGDPVCNACGLYYKLHSVNRPLTMKKDGIQTRKRKPKSSQQIQPMNGLTTGKG
FDCRAGGIGVQSNRVVFCRQATTFDDTVPVSLDRDRTKATERASVPSGADIPADGAAQQLAGTGPAVRGQL
TRSARWRRWQW

ZF1: ~180NT, 60 A 992202-992381
<u>AAPPEHRECVNCGSSDTPLWRRDIVGHTLCNACALYTRQNPGTNRPPNRSQKAKQTVVSG</u>
ZF2+CD: <492 NT, 164 AA 992463-992954
*TPPAQGNRRSGVTCANCQTTTTTLWRRNNQGDPVCNACGLYYKLH***SVNRPLTMKKDGIQTRKRKPKSSQQI**
QPMNGLTTGKGFDCRAGGIGVQSNRVVFCRQATTFDDTVPVSLDRDRTKATERASVPSGADIPADGAAQQL
AGTGPAVRGQLTRSARWRRWQW

>AgamGATA456bba *Drosophila Pannier ortholog*
MDFQFGEGRECVNCGAISTPLWRRDGTGHYLCNACGLYHKMNGMNRPLIKPSKRLVSVSIQFPYHMQTATR
RLGLCCTNCGTRTTTLWRRNNDGEPVCNACGLYFKLHGVNRPLAMRKDGIQTRKRKPKKTGGSGGSADVMA
L

ZF1: (Chr2R:1033129-1033326)
MDFQFGEGRECVNCGAISTPLWRRDGTGHYLCNACGLYHKMNGMNRPLIKPSKRLVSVSIQFPYHM
ZF2+CD: (Chr2R:1034205-1034435)
*QTATRRLGLCCTNCGTRTTTLWRRNNDGEPVCNACGLYFKLH***GVNRPLAMRKDGIQTRKRKPKKTG**GSGGS
ADVMAL

<u>Additional GATA456 on Chromosome 3 (NW_045838.1)</u>

>AgamGATA456bbb *Drosophila GATAd ortholog*
*NSQQKDMSCTNCGTTTTTIWRRNIRGEMVCNACGLYFKLHGV***NRPHTMRRDTIHTRRRR**

ZF2+3'CDS:(Chr3:2756384-2756187)
*NSQQKDMSCTNCGTTTTTIWRRNIRGEMVCNACGLYFKLHGV***NRPHTMRRDTIHTRRRR**

<u>*Anopheles* GATA123 on Chromosome 2R (NW_045682.1)</u>

>AgamGATA123 *Drosophila Grain ortholog*
TEGRECVNCGATSTPLWRRDGTGHYLCNACGLYYKMNGQNRPLIKPKRRLSAARRAGTSCANCKTTTTTLW
RRNQGGEPVCNACGLYYKLHNVSNRPLTMKKEGIQTRNRKLSSKSKKKKGIPGSCLPLGGHLGDLMKPLDH
KPSFPGAFPGSMGKLTNIKCLLYKFNNQKNIIDLKKKPLKLINYNFDCIILRFAHSTGQHSHLSGGLHPAH
THMHGGWYTTGMGALGTSGGLQNGFGGAGSLGGGVVPHSQSYHLGLNSMVSTLF

ZF1: (Chr2R:2689570-2689421)
<u>TEGRECVNCGATSTPLWRRDGTGHYLCNACGLYYKMNGQNRPLIKPKRRL</u>
ZF2: (Chr2R:2664725–2664594)
SAARRAGTSCANCKTTTTTLWRRNQGGEPVCNACGLYYKLHNVS
3'CD: (Chr2R:265727202656754)
NRPLTMKKEGIQTRNRKLSSKSKKKKGIPGSCLPLGGHLGDLMKPLDHKPSFPGAFPGSMGKLTNIKCLLY
KFNNQKNIIDLKKKPLKLINYNFDCIILRFAHSTGQHSHLSGGLHPAHTHMHGGWYTTGMGALGTSGGLQN
GFGGAGSLGGGVVPHSQSYHLGLNSMVSTLF

Daphnia pulex **(water flea, crustacean)**

DpulGATA456 cluster at 91K-129K on Scaffold 32 (JGI060905:Scaffold_32)

>DpulGATA456ba *Drosophila Serpent ortholog*
DGRECVNCGSISTPLWRRDGTGHYLCNACGLYHKMNNGTQRPLIKQTRRLSTTRRLGLRCANCATTTTSLW
RRNNQGETVCNACGLYFKLHGVNRPLAMKKDNIQTRKRKRKGDTTPVNKVVL

ZF1: (Sca32:91715-91893)
DGRECVNCGSISTPLWRRDGTGHYLCNACGLYHKMNNGTQRPLIKQTRRL
ZF2: (Sca32:92099-92263)
*STTRRLGLRCANCATTTTSLWRRNNQGETVCNACGLYFKLH***GVNRPLAMKKDNI**
3'CD: (Sca32:92342-92729)
QTRKRKRKGDTTPVNKVVLGAAPASSSGSSGSAGHSGHHHHLSQHHHINSNQGQQQQGLTSSSSLANHLNP
GSPTNNSYGAPISMMMGNNGSGKLVYQQHHQHHHQDPANSISPIHHSDLIKTEQHSVLN

>DpulGATA456a *Drosophila GATAe ortholog*
MLEGRECANCSAIATPLWRRDGNNHYLCNACGLYKLTNGTNRPPVRQPPSSGNRRAGLTCSNCNTSTTTLW
RRNANGEPVCNACGLYFKLHNVSVCRPLAMKKEGIQTRKRKPKAGTPSGAPTEKSSNKRSKNSNNTSGGQL
ILQHPSGSSGSQLHQQHHAQLNQHYEEMKHELGETDDHEMTTISSQMLGVGGASAVIHYTPTVITSSSLTV
GNGGAVVLMEPSGLITTVTSNGAEDEDQQQHHHHHHHHQADGGNDEASASNNGDHSPIHLPSTAFLNTHIS
NLPPLEPVVMMHGSTLVTRGGSPIGNDVGSNIVIRQFERQVD*

ZF1: (Sca32:100254-100403)
MLEGRECANCSAIATPLWRRDGNNHYLCNACGLYKLTNGTNRPPVRQPPS
ZF2: (Sca32:100591-100722) (Shifted 2nd ZF/3'CD boundary)
SGNRRAGLTCSNCNTSTTTLWRRNANGEPVCNACGLYFKLHN
3'CD: (Sca32:106460-106565)
VCRPLAMKKEGIQTRKRKPKAGTPSGAPTEKSSNK
3' exon: (Sca32:106631-106801) (from EST)
SKNSNNTSGGQLILQHPSGSSGSQLHQQHHAQLNQHYEEMKHELGETDDHEMTTIS
3' exon: (Sca32:106916-107210) (from EST)
SQMLGVGGASAVIHYTPTVITSSSLTVGNGGAVVLMEPSGLITTVTSNGAEDEDQQQHHHHHHHHQADGGN
DEASASNNGDHSPIHLPSTAFLNTHIS
3'exon3: (Sca32:108039-108165) (from EST)
NLPPLEPVVMMHGSTLVTRGGSPIGNDVGSNIVIRQFERQVD*

>DpulGATA45bb *Drosophila Pannier ortholog*
SYLEGRECVNCGSISTPLWRRDGTGHYLCNACGLYHKMNNGINRPLLKPPRRLSATRRLGLCCTNCGTTTT
TLWRRNAEGEPVCNACGLYHKLHGVNRPLAMRKDGIQTRKRKPKSNSAAAAAAAAVAAAVAANHTNSVAAG
AASMDHQHSSSIGGGISSHHHHHHHHPHQHNGSSSSSANHLIGGMKIDRMQSDLHSKSTERGMIPPTIAHL
KSFRDFRVAAD

ZF1: (Sca32:118388-119816)
SYLEGRECVNCGSISTPLWRRDGTGHYLCNACGLYHKMNNGINRPLLKPPRRL
ZF2+CD: (Sca32:118989-119181) fused 2nd ZF/3'CD exons
*SATRRLGLCCTNCGTTTTTLWRRNAEGEPVCNACGLYHKLH***GVNRPLAMRKDGIQTRKRKPKSNSA**AAAAA
AAVAAAVAANHTNSVAAGAASMDHQHSSSIGGGISSHHHHHHHHPHQHNGSSSSSANHLIGGMKIDRMQSD
LHSKSTERGMIPPTIAHLKSFRDFRVAAD

Daphnia GATA123 on JGI060905:Scaffold 35

>DpulGATA123 *Drosophila Grain ortholog*
TKEGRECVNCGATSTPLWRRDGTGHYLCNACGLYYKMNGQNRPLIKPKRRLSAARRAGTTCANCKTTMTTL
WRRNHNGEPVCNACGLYYKLHNVQVNRPLTMKKEGIQTRNRKLSSKSKKKKG

5’Exon (Sca35:161663-160945)
MAPHHPHHAHHHHHHPAHHPSAWSPFAASAVAAQAAQQQQQQSSTSGGSSSSSSSGTAKHPHSSPHLLTFP
PTPPEDAASESAAAAAAAAAAAATAAANQEFQQQQQQQQQQQQERNGNHSGSVAMDLAGGSASPVDNKSSL
SSSFSCFSSPSDLKMMSGFNNSGSGENHKPSGQHGSGGANASSVSGLMFAAAGFAAGSSFPACSVAGKPRE
GSHSPVNSNNNNNGNAFAIPASASPAS
5’Exon (Sca35:160875-160586)
LPPPPLTPHQSGGGQQQGSGGANSAGSYSYGGSTPTAADYSAAAVAAYGAAGFHSAVHHHSHAHHQQSGSV
FSAKSLHHHHHHQSARPRTKTRSSA

ZF1: (Sca35:159013-158868)
EGRECVNCGATSTPLWRRDGTGHYLCNACGLYYKMNGQNRPLIKPKRRL
ZF2: (Sca35:156330-156199)
SAARRAGTTCANCKTTMTTLWRRNHNGEPVCNACGLYYKLHNV
3’CDS: (Sca35:146988-146455)
VNRPLTMKKEGIQTRNRKLSSKSKKKKG
3’exons: (Sca35:125447-125467)
SISSSASASASAGCHLSSYQDVMSMGAGSALDKAAVAAAAAAAAGFPGFAAAAAVASAAGASSAMSHYAVYAG
NGQMHHSAHHHPHHAGLNHSAAAQMGGFHHHGSPSGPPPGSMTIHHSMGGISSSALGLGSTF

Ixodes scapularis **(chelicerate, tick)**

3 *Ixodes* GATA456 genes

>IscaGATA456ba *Drosophila Serpent ortholog*from cDNA G893P513FD21.T0
MGVIVTIDSFGRIPGSQVNGMSHQYGQYLGFEHGGQATAVQWSHGMDASGAAMTGLTSYTALVGDKRASLA
DLEYFGGEGRECVNCGAISTPLWRRDGTGHYLCNACGLYNKMNGAHRPIIKTPRRLSASRRVGLTCSNCET
STTSLWRRNNVGEPVCNACGLYFRLHGVNRPLAMKKDSIQTRKRKPKNSSSGSGSSGVKGLENPGSVGGVS
GLGLSLSSGGVLVGSGSLEERQPQHHPAQQQGADPWHHSHPRTVLSRHERAAEQERSAAAHQPGSAVQLQP
GFPDLQRRAPVY*

5'CDS: no trace found
MGVIVTIDSFGRIPGS
5'CDS: from trace (BIdea45ec960a49fcb5e4596f58fb7cc3d.F.6.T0)
QVNGMSHQYGQYLGFEHGGQATAVQWSHGMDASGAAMTGLTSYTALVGDKRASL
ZF1: from trace (1105499454364)
ADLEYFGGEGRECVNCGAISTPLWRRDGTGHYLCNACGLYNKMNGAHRPIIKTPRRL
ZF2: from trace (1100737814541)
SASRRVGLTCSNCETSTTSLWRRNNVGEPVCNACGLYFRLHGV
3'CD: from trace (1103668122187)
NRPLAMKKDSIQTRKRKPKNSSSGSGSSGVKGLENPGSVGGVSGLGLSLSSGGVLVGSGSLEERQPQHHPA
QQQGADPWHHSHPRTVL
3'CDS: from trace (1100024400230)
SRHERAAEQERSAAAHQPGSAVQLQPGFPDLQRRAPVY*

>IscaGATA456a *Drosophila GATAe ortholog* assembled using tracembler
EYAGFGEARECVNCGAISTPLWRRDGTGHYLCNACGLYHKMNGSTRPIVKPQRRMVS
QQQPSVSRRVGMCCSNCGTTTTTLWRRNNEGEPVCNACGLYFKLHNVSPSSAAYK

ZF1: from trace (1101298794630)
EYAGFGEARECVNCGAISTPLWRRDGTGHYLCNACGLYHKMNGSTRPIVKPQRRMVS
ZF2: from trace (1101347043341)
QQQPSVSRRVGMCCSNCGTTTTTLWRRNNEGEPVCNACGLYFKLHNV

>IscaGATA456bb *Drosophila Pannier ortholog*
DAERLQEFYSNESKECVNCGAISTPLWRRDCTGHYLCNACGLYSKMNGANRPLMRPPKRPVRSQ

ZF1: from trace (1101170759193)
DAERLQEFYSNESKECVNCGAISTPLWRRDCTGHYLCNACGLYSKMNGANRPLMRPPKRPVRSQ

>IscaGATA456partial: 3' CD only (not clear if in GATA456a or GATA567bb)
VNRPLAMKKEGIQTRKRK

3'CD: from trace G800P65919RO6.T0
VNRPLAMKKEGIQTRKRK

One *Ixodes* GATA123 gene

>IscaGATA123 from cDNA G894P56RC12.T0
PGVLAQEDMDMFFHSLDGSPGSSYYSNPAARAVHGYRPSHPRVPGGQVCRPHSHSALHPWISDPAKAAAAA
AAAMVPHHGPASWCSPFAGKPQPSPPTAPSHSSPHLFSFPPTPPKDATPDPAAAEAPGYACDGDHKPGGML
TPLAASSACGKREGTFPASSPPYPHYVPHPGSELAGYHGFHAGPLQLAKAPLPPPAGYGQAAKPRTKGRSS
AEGRECVNCGATSTPLWRRDGTGHYLCNACGLYHKMNGQNRPLIKPKRRLSAARRAGTSCANCKTTTTTLW
RRNQNGEPVCNACGLYFKLHNVNRPLTMKKEGIQTRNRKLSSKSKKKKGLTMSLPDCIKPLDKGFSSFSPA
GHQFASSMSMTMNHYMHQGNMNMPISMGGTFVTSQHMGSAAGLSGLGLGPSASAGLGLAASNSMVGAMA

ZF1: from trace (1101127246305)
AEGRECVNCGATSTPLWRRDGTGHYLCNACGLYHKMNGQNRPLIKPKRRLVSTPS

ZF2: from trace (1101271985896)
SAARRAGTSCANCKTTTTTLWRRNQNGEPVCNACGLYFKLHNV

3’CD:from trace (1446667586)
VNRPLTMKKEGIQTRNRKLSSKSKKKKGLTMSLPDCIKPLDKGFSSFSPAGHQFASSMSMTMNHYMHQGNM
NMPISMGGTFVTSQHMGSAAGLSGLGLGPSASAGLGLAASNSMVGAMA

Lottia gigantea **(mollusk, limpet)**

Lottia GATA456 cluster on Scaffold 67, within 45 KB region

>LgigGATA456Na
HDYFADLEGRECVNCGAISTPLWRRDGTGHYLCNACGLYHKMNGINRPLIKPQRRLVSASRRMGLSCSNCH
TSTTTLWRRNNEGEPVCNACGLYYKLHGVIHRPLAMKKDGIQTRKRKPKSKERSPHKTSEKSG
ZF1: (Sca67:1028598-1028768)
DYFADLEGRECVNCGAISTPLWRRDGTGHYLCNACGLYHKMNGINRPLIKPQRRL
ZF2: (Sca67:1029429-1029557)
SASRRMGLSCSNCHTSTTTLWRRNNEGEPVCNACGLYYKLHGV
3’CD:(Sca67:1029871-1029972)
HRPLAMKKDGIQTRKRKPKSKERSPHKTSEKSG

>LgigGATA456Nc
LFISANDDFYSEGRECVVCGAMSTPLWRRDGTGHYICNACGIYHKMAANGRPESKPNLTENRTVSLGFSSR
RMGLACANCMTSTTTLWRRNAEGEPVCNACGLYYKLHQVRKCHVLVNRPMSMKKDGIQTRKRKPRTPSKLK
TSPKDNQQRHVPSEPTQTLPPLSLPSSHQGALHQHRTNQSQGLLDLTLQRSEAHQGSSF
ZF1: (Sca67:1056302-1056508)
LFISANDDFYSEGRECVVCGAMSTPLWRRDGTGHYICNACGIYHKMAANGRPESKP
ZF2: (Sca67:1058155-1058295)
SRRMGLACANCMTSTTTLWRRNAEGEPVCNACGLYYKLHQV
3’CD: (Sca67:1058425-1058680)
NRPMSMKKDGIQTRKRKPRTPSKIK

>LgigGATA456Nb
DYYADLEGRECVNCGALSTPLWRRDGTGHYLCNACGLYHKMNSVNGAILKPQRRLSASRRIGLSCSNCHTS
TTTLWRRNNEGEPVCNACGLYYKLHGVINRPLAMKKDGIQTRKRKPKGQGKTKSSSKSDSVGKPSSFDYYL
KK
ZF1: (Sca67:1072508-1072371)
DYYADLEGRECVNCGALSTPLWRRDGTGHYLCNACGLYHKMNSVNGAILKPQRRL
ZF2: (Sca67:1072895-1072767)
SASRRIGLSCSNCHTSTTTLWRRNNEGEPVCNACGLYYKLHGV
3’CDS (Sca67:1073063-1073230)
NRPLAMKKDGIQTRKRK

Lottia GATA123 ortholog on JGI scaffold 833

>LgigGATA123
FFSEGRECVNCGATSTPLWRRDGTGHYLCNACGLYHKMNGSNRPLIKPKRRLSAARRAGTSCSNCGTSTTT
LWRRNHNGDPVCNACGLYYKLHNVSNRPLTMKKDGIQTRNRKMSTKSKKGKKGMNCMSDFLKPLDKQFGGF
GSPNFNHAMHAPMPGYNYMTGGSSFGGGFMSSGGHQMMGSLSSGFNNFPTSGSFNSSFSGIPSSGLNLTTA
TNMVGAAMA

ZF1: (LotScaf24:2777006-2777161)
FFSEGRECVNCGATSTPLWRRDGTGHYLCNACGLYHKMNGSNRPLIKPKRRL
ZF2: (LotScaf24:2782983-2788585)
SAARRAGTSCSNCGTSTTTLWRRNHNGDPVCNACGLYYKLHNVS
3’CD: (LotScaf24:2788208-2788585)
NRPLTMKKDGIQTRNRKMSTKSKKGKKGMNCMSDFLKPLDKQFGGFGSPNFNHAMHAPMPGYNYMTGGSSF
GGGFMSSGGHQMMGSLSSGFNNFPTSGSFNSSFSGIPSSGLNLTTATNMVGAAM

Capitella capitata **(annelid, polychaete)**

Two linked GATA456 genes on Scaffold 218 (JGI:Scaffold218)

>CcapGATA456Nb
QSDYFCEGRECVNCGAMSTPLWRRDGTGHYLCNACGLYHKMNGLNRPMMKPQKRMASRRMGLQCANCQTST
TTLWRRNADGEPVCNACGLYFKLHGV*HRPMSMRKDGIQTRKRKPKGPSKVADGQCHLNLTVLSLYMRFFIF*
QIHHV

ZF1: (Sca218:22823-22987)
QSDYFCEGRECVNCGAMSTPLWRRDGTGHYLCNACGLYHKMNGLNRPMMKPQKRM
ZF2+3'CDS: (Sca218:23099-23374)
*ASRRMGLQCANCQTSTTTLWRRNADGEPVCNACGLYFKLHGV**HRPMSMRKDGIQTRKRKPKGPSKV**ADGQC*
HLNLTVLSLYMRFFIFQIHHV

>CcapGATA456Nc
LCPFTCSLFRRFMAAADHLLKPLLPPAHPAHRKPVLPLEDVDLFFRALNPSTSAAIATEATAAYGQQSLGS
AAAGARLAPSPWAPDGSMLAATQSSMHSLGGHGSPPAYMHADTAGAAASSFLYPATPSPVYVPTTRAVLPQ
YGGGGSGVVSSTPSAHQPPASSVWPPPLQSSPETPSAYGSGAPHRFGPYSSNPSPPGLQTHMGRSGPATDG
LGSPLHARSAASTGLSSYGYMHSGDPLQAAWSGLNASGMLHGQQTSPLRRSSLGQYMHPLSDRHIVHLTSS
FPVQYCLVTSRQAETDYYGSPAMEGGVGRECANCGSTYAPLWRWNGTGHLLCNACGVHVMSGFAKPVMKTS
GGRRVSVSRRVGLSCANCHTSTTTLWRRNNEGEPVCNACGLYFKLHGVNRPMSMKKEGIQTRKRKPKGSGK
QKSSPSHSKGTL

5'CDS: (Sca218:31331-32218)
LCPFTCSLFRRFMAAADHLLKPLLPPAHPAHRKPVLPLEDVDLFFRALNPSTSAAIATEATAAYGQQSLGS
AAAGARLAPSPWAPDGSMLAATQSSMHSLGGHGSPPAYMHADTAGAAASSFLYPATPSPVYVPTTRAVLPQ
YGGGGSGVVSSTPSAHQPPASSVWPPPLQSSPETPSAYGSGAPHRFGPYSSNPSPPGLQTHMGRSGPATDG
LGSPLHARSAASTGLSSYGYMHSGDPLQAAWSGLNASGMLHGQQTSPLRRSSLGQYMHPLSDRHIVHLTSS
FPVQYCLVTSRQ
ZF1: (Sca218:33124-33309)
AETDYYGSPAMEGGVGRECANCGSTYAPLWRWNGTGHLLCNACGVHVMSGFAKPVMKTSGGRRV
ZF2: (Sca218:33372-33500)
SVSRRVGLSCANCHTSTTTLWRRNNEGEPVCNACGLYFKLHGV
3'CDS: (Sca218:33555-33662)
NRPMSMKKEGIQTRKRKPKGSGKQKSSPSHSKGTL

Two addtional GATA456 genes on JGI Scaffolds 782 and 272

>CcapGATA456Na
DYYVEGRECVNCGAMSTPLWRRDGTGHYLCNACGLYHKMNGVNRPLVKPQRRIQGSRRLGLQCANCSTTTT
TLWRRNNEGEPVCNACGLYYKLHQVARPISMKKDGIQTRKRKPKGSGKSKSKHKQDNSSSGIT

ZF1: (Sca782:47209-47036)
DYYVEGRECVNCGAMSTPLWRRDGTGHYLCNACGLYHKMNGVNRPLVKPQRR
ZF2: (Sca782:48942-46811)
IQGSRRLGLQCANCSTTTTTLWRRNNEGEPVCNACGLYYKLHQV
3'CDS: (Sca782:46757-46644)
ARPISMKKDGIQTRKRKPKGSGKSKSKHKQDNSSSGIT

>CcapGATA456Nd
SSQAKYRPFPLSGHTCTNCGTTKTTTWRRNLTGEPLCNACGCYLKLHKVSIGSTLFLPLRMASSSSRETHN
CANCGTQQTTMWRRNTDGEPVCNACGLYFKLHKVNRPLSMVKGEIQSRRRKPKSSSSPGIMPLCSILPKAE
PQLPQEGSASPLPPPPPTEGVVRLPTSTPVNP

ZF1: (Sca272:27339-27208)
SSQAKYRPFPLSGHTCTNCGTTKTTTWRRNLTGEPLCNACGCYLKLHKVSIGSTLFLPLRM
ZF2: (Sca272:27663-27161)
ASSSSRETHNCANCGTQQTTMWRRNTDGEPVCNACGLYFKLHKV
3'CD: (Sca272:27663-26850)
NRPLSMVKGEIQSRRRKPKSSSSPGIMPLCSILPKAEPQLPQEGSASPLPPPPPTEGVVRLPTSTPVNP

Capitella GATA123 ortholog on JGI scaffold 833

>CcapGATA123
SSEGRECVNCGATSTPLWRRDGTGHYLCNACGLYHKMNGQNRPLIKPKRRLVSTRLWRRNHNGDPVCNACG
LYYKLHNVSSAARRAGTNCANCGTTTTTLWRRNHNGDPVCNACGLYYKLHNVSNRPLTMKKDGIQTPNPKM
S

ZF1: (Sc833:14048-13863)
SSEGRECVNCGATSTPLWRRDGTGHYLCNACGLYHKMNGQNRPLIKPKRRLVSTRLWRRNHNGDPVCNACG
LYYKLHNVS
ZF2: (Sc833:13326-13198)
SAARRAGTNCANCGTTTTTLWRRNHNGDPVCNACGLYYKLHNVS
3'CDS (Sc833:9902-9844)
NRPLTMKKDGIQTPNPKMS

Schmidtea mediterranea **(flatworm, planarian)**

Schmidtea mediterranea-3.1-supercontigs

>SmedGATA456Na (contig 2459)
ZF1
EGRECVNCGAVNTPLWRRDGQGNYLCNACGLYQKMNGQNRPLIKPKRRL
ZF2
SASRRTGTICSNCNTSTTTLWRRNSNGEPVCNACGLYFKLHSVS
3'CD
VSRPPTMKKEGIQTRNRK

>SmedGATA456Nb (contig 7909)
ZF1
RECVNCGASNTQLWSRDNSGYYLCDECDRFSQNNSRNLEKL
ZF2
KRSDLECSNCKITKTSLWRRNNEGEPVCNACGLYYKLHKV
3'CD
RPLSMRKEGIQTRKRK

>SmedGATA456Nc (contig 45)
ZF1
KECVICGMENGDTYHQDGNGNFHCLNC
ZF2
AKRTGLQCSNCKTENTTLWRRNSEGQPVCNACGLYYRLHKV
3'CD
HRPPTMRKEILQSRKRK

>SmedGATA123 (contig 2203)
ZF1
DMSTEGRECVNCGATQTPLWRRDGTGHYLCNACGLYHKMNGTNRPLIKPKRRL
ZF2
SSARKVGTTCINCGTNHTTLWRRNQQGDSVCNACGLYYKLHHV
3'CD
RPISMKKDLIQTRNRK

Drosophila melanogaster **(insect, fruit fly)**

GATA456 cluster at 11819K-11866K on Chromosome 3R (NT_033777.2)

>DmelSerpent NM_169691.1(A-isoform), NM_169694.1 (B-isoform)
NM_001032019.1 (D-isoform)

5'CDS: B isoform (Chr3R:11819864-11821446)
MTKTTKPKEKAAAGGAVIGSGSGLGSVTKAGGGSLLSNAADSKIRTAKSNNNKRQAGRAATALAATTTAS
ALAATTTAGATGSNAAANETEIAIETENGEAATPTAAATAAAANLSSLESARSQALTSVVSETARQAVTT
ANASATSTSTSTVTAATEIATATASDTAATSEAAIDDDPSAINTNNNNNNSKAQNDASESVKTKVISYHQSE
DQQQQQQQQAQIYEQQQQFLSQQLISHHQQEQHQQAQQQQHQQVVQEQHQASWLAYDLTSGSAAAAAAAA
AAASHPHLFGQFSYPPSHHTPTQLYEHYPSTDPIMRNNFAFYSVYTGGGGGVGVGMTSHEHLAAAAAAAA
AVAQGTTPNIDEVIQDTLKDECFEDGHSTDYHVLTSVSDMHTLKDSSPYALTHEQLHQQQHHHQQQLHHH
QQQQQQLYHQQQQQQQQQQHHHHHNNSTSSAGGDSPSSSHALSTLQSFTQLTSATQRDSLSPENDAYFAA
AQLGSSLQNS
5'CDS A isoform, beginning overlaps with B (Chr3R:11819864-11821446)
MTKTTKPKEKAAAGGAVIGSGSGLGSVTKAGGGSLLSNAADSKIRTAKSNNNKRQAGRAATALAATTTASA
LAATTTAGATGSNAAANETEIAIETENGEAATPTAAATAAAANLSSLESARSQALTSVVSETARQAVTTAN
ASATSTSTSTVTAATEIATATASDTAATSEAAIDDDPSAINTNNNNNNSKAQNDASESVKTKVISYHQSEDQQ
QQQQQQAQIYEQQQQFLSQQLISHHQQEQHQQAQQQQHQQVVQEQHQASWLAYDLTSGSAAAAAAAAAAAS
HPHLFGQFSYPPSHHTPTQLYEHYPSTDPIMRNNFAFY
5'CDS A,B isoform, end contain D-isoform (Chr3R:11825048-11825495)
SVYTGGGGGVGVGMTSHEHLAAAAAAAAAVAQGTTPNIDEVIQDTLKDECFEDGHSTDYHVLTSVSDMHTL
KDSSPYALTHEQLHQQQHHHQQQLHHHQQQQQQLYHQQQQQQQQQQHHHHHNNSTSSAGGDSPSSSHALST
LQSFTQLTSATQRDSLSPENDAYFAAAQLGSSLQNSSVYAGSLLTQTANGIQYGMQSPNQTQAHLQQQHHQ
QQQQQHQQHQQQQLQQQQQQHHHNQHQHHNSSSSSPGPAGLHHSSSSAATAAAVAAATAAVNGHNSSLEDG
YGSPRSSHSGGGGGGTLPAFQRIAYPNSGSVERYAPITNYRGQ
5'CDS: Isoform D only (Chr3R:11825048-11825495)
MQSPNQTQAHLQQQHHQQQQQQHQQHQQQQLQQQQQQHHHNQHQHHNSSSSSPGPAGLHHSSSSAATAAA
VAAATAAVNGHNSSLEDGYGSPRSSHSGGGGGGTLPAFQRIAYPNSGSVERYAPITNYRGQ
5'CDS: Isoforms A, B, D (Chr3R:11825568-11825781)
NDTWFDPLSYATSSSGQAQLGVGVGAGVVSNVIRNGRAISAANAAAAAAADGTTGRVDPGTFLSASASLSA
ZF1: Isoform B only (Chr3R:11826335-11826507)
TLFDADYFTEGRECVNCGAISTPLWRRDNTGHYLCNACGLYMKMNGMNRPLIKQPRRL
5'CDS: Isoforms A, D (Chr3R:11826911-11827128)
MAAESGGDFYKPNSFNVGGGGRSKANTSGAASSYSCPGSNATSAATSAVASGTAATAATTLDEHVSRANSR
RL
ZF2: Isoforms A,B,D (Chr3R:11827474-11827638)
*SASKRAGLSCSNCHTTHTSLWRRNPAGEPVCNACGLYYKLH***SVPRPLTMKKDTIQ**
3'CD: Isoforms A,B,D (Chr3R:11827892-11828045)
KRKRKPKGTKSEKSKSKSKNALNAIMESGSLVTNCHNVGVVLDSSQMDVND
3'CDS: Isoforms A,B,D (Chr3R:11828124-11829807)
DMKPQLDLKPYNSYSSQPQQQLPQYQQQQQLLMADQHSSAASSPHSMGSTSLSPSAMSHQHQTHPHQQQQQ
QLCSGLDMSPNSNYQMSPLNMQQHQQQQSCSMQHSPSTPTSIFNTPSPTHQLHNNNNNNNNSSIFNNNNNN
NSSSNENNNKLIQKYLQAQQLSSSSNSGSTSDHQLLAQLLPNSITAAAAAAAAAAAAIKTEALSLTSQAN
CSTASAGLMVTSTPTTASSTLSSLSHSNIISLQNPYHQAGMTLCKPTRPSPPYYLTPEEDEQPALIKMEEM
DQSQQQQQQQQHQQQQHGEIMLSRSASLDEHYELAAFQRHQQQQQQLQQQTAALLGQHEQHVTNYAMHKFG
VDRETVVKME

>DmelGATAe NM_142259.2(+)
5’CDS:(Chr3R:11838461-11838505)
MVCKTISPSVNMQLK
5’CDS:(Chr3R:11841950-11842287)
MEQQTTQQQQQQQQQQQQQQQLQQQQHQALTKQQLQLLDKIKLESSNGADQLAQQTANNLDEQQEQQQQHQ
QQAATSVGVVVQTGQAGVSEPEEQYVVVPRNQRRILTTAGTL
5’CDS:(Chr3R:11842431-11843364)
ELNEAREGEPSTNASNASSGSASDSHIEYQRSAHQSPGATHYVQMAPRNAEVTEQVGAAAGAPPGTIFAYP
IICNGDDVAAIKIETLEKGEATGESQQQQQQLQQHQHQQQQQCPTPNGASYGETIVISSEAEALQHHHQQQ
QQHQQQQHQQQHHQHQAAAAASAAAQTVHIATSSHGGTVRFVTEDVRFTTAGPETSASNMYYDVPVVDGSV
HANESKTYADLGNAYAPFPPSSSFSSNSYAATLQQGNTIYSVPGTGQFLAKSESGLNQTGLLRQTGPATFQ
TISFEGGNGIEPLWASPAPPEYQSVQF
ZF1+ZF2+3’CDS:(Chr3R:11843433-11843928)
SNFHPQVIDEYGSGNMSTSHWPPASSIGQYDGSLVTASSTSSPNHELKCENCHGPFLRKGSEYFCPNCPAF
MRMAPRITQRQAKPKA*AAAPNNRRNGVTCANCQTNSTTLWRRNNEGNPVCNACGLYYKLH***NMNRPLSMKKE**
GIQKRKRKPKNNGGAPMHRAPLP
3’CDS: (Chr3R:11843987-11844317)
SMSQGVNLMANSPLYPSQVPVSMLNSQLNSQQNSSPELHDMSTTGQAGGQRVVSISLNATAPPTPDGTLNM
SARHHVTGESHSPYSQQSTPQSQSPHLPGTVPINRQIVQP
3’CDS: (Chr3R:11844379-11844475)
VPTIESSRSSNTELTPSVITRTGLPERSSNN

>DmelPannier PnrA isoform NM_057337.2
PnrB isoform NM_169697.2
CDS1: A isoform
MGILLSDGD
ZF1: A isoform (Chr3R:11860802-11860828)
STSDQQSTRDYPHFSGDYQNVTLSAASASTSASASATHVAAVKMYHSSAVAAYTDLAAAGSAASAGVGVGV
SGYHQQAVNAPVYVPSNRQYNHVAAHFGSAAAQNAWTTEGFGSAHAQFYSPNAAVMMGSWRSAYDPSGFQR
SSPYESAMDFQFGEGRECVNCGAISTPLWRRDGTGHYLCNACGLYHKMNGMNRPLIKPSKRLVSA
ZF1: B isoform (Chr3R:11864438-11864929)
MYHSSAVAAYTDLAAAGSAASAGVGVGVSGYHQQAVNAPVYVPSNRQYNHVAAHFGSAAAQNAWTTEGFGS
AHAQFYSPNAAVMMGSWRSAYDPSGFQRSSPYESAMDFQFGEGRECVNCGAISTPLWRRDGTGHYLCNACG
LYHKMNGMNRPLIKPSKRLVSA
ZF2+3’CD: A, B isoforms (Chr3R:11864992-11865268)
*TATRRMGLCCTNCGTRTTTLWRRNNDGEPVCNACGLYYKLH***GVNRPLAMRKDGIQTRKRKPKKTGSGSAVG**
AGTGSGTGSTLEAIKECKEEH
3’CDS: A, B isoforms (Chr3R:11865337-11866034)
DLKPSLSLERHSLSKLHTDMKSGTSSSSTLMGHHSAQQQQQQQQQQQQQQQQQQQQSAHQQCFPLYGQTTT
QQQHQQHGHSMTSSSGQAHLSARHLHGAAGTQLYTPGSSSGGGSASAYTSHSAETPALSNGTPSPHYQHHH
HLGGTHGHHVTAAAAHHHFHAAAAVAAYGVKTEASATNYDYVNNCYFGGTFGALGGAATTTAMAGGAASEL
AGYHHQHNVIQAAKLMATS

Additional GATA456 gene on Chromosome 2L (NT_033779.4)

>DmelGATAd (NM_135539.2)
5'CD: (Chr2L:10333914-10334812)
MNNVPHKFRQVCRLCLTLVNECDVAVLQIYDNSSHNNSAPDRISADFRGVANRANCFCSALSPNPCSCIVD
NPLSANKNERRNQGTPVPVPIPIPVSVPVPVPSQQIQSQTLHHHQNQTKIHYTPSVPSAADAKSSERERQC
QLESDIAERSTVFKSQSSSDHEHIEYSGETPSPSASLSQSQTKSEPMREEEEHQHNHNQQSGREETSVNTT
NISGTDNYGKEHGRDDSSSPHLTFQIFNCLSIK
5'CD: (Chr2L:10335781-10335924)
ALPNDGLPNVVCGDCRQKLDSFEKFRTMAHNSQIALKEFLNISKNLRP
5'CD: (Chr2L:10336022-10336883)
DPNDLETKLDAILKASSEAIAAKALTELSTFSKVKYDHSKLDSSIQPGLDRKIEISNQEMPAHSLYPSLFK
SITMKSDQKSKAMSGSSHSQFDSKLQKDSDIEKYENLQQQLETAAVLMDISKKIVISPPCSNPQSPCFSAA
VDTSIKSSVIKSKRPSNQNEIQDGVEIDLSVKKQKNDYSNQRNAAPIHHFCQTPMLDIQSHLRSEEDFQNY
SITINQVGGSDFKSKAPKASTGSLLDSGDSSDSHKLEMDITSSINDRKTPDSLSSDHATDAATTQLWQALA
RSA
5'CD: (Chr2L:10336945-10337072)
AKSKEDNPASQIFRNMMSQPFAFPVPSTVSFTKVPEEPIALLK
5'CD: (Chr2L:10337141-10337293)
DLSEAQSSKSKPCRRKQSFPTKTDCIDVVNENVTDTYTTSEATPDDKKDKR
ZF2+3'CD: (Chr2L:10337368-10337596)
NINLFNAIP*GAQKDMSCSNCGTLTTTIWRRSVRGEMVCNACGLYFKLHGV***NRPHSMRRDTIHTRRRRPKEL**
ERSKK
3'CD: (Chr2L:10338019-10338116)
KHKQMSSCSSIETTKQDFLTARESLAISGLVLN
3'CD: (Chr2L:10338232-10338456)
KFKKEIDDTETPAAAALKDILSRRKKSNSLPAFNDTCESADLSAPLNLVSSENNAKLT

Drosophila GATA123 on Chromosome 3R (NT_033777(-))

>DmelGrain (NM_169206.1)
5'CDS_1: (Chr3R:4005077-4005398)
MDMTSTAEAAARSWYDSPRLGGGGSSGGGNGGGVSPQTNGLGSAGSSLAHSHHSLSSGASSAGS
SVGVGSALGGGGGSGLDTSDMSAFYALESNGHHRRYYPSYHQH
5'CDS_2: (Chr3R:3993553-3993839)
TSRMPSTHASPQVCRPHFHTPLSPWLTSEHKSFAPASAWSMGQFACPQEPQVEHKLGQMGQSHQT
TAAGQHSFPFPPTPPKDSTPDSVQTGPSEYQ
5'CDS_3: (Chr3R:3993093-3993252)
AVMNAFMHQQATGSTSLTDASCALDIKPSIQNGSASGSSGSGTTHTSTPKQRE
ZF1: (Chr3R:3986685-3986830)
EGRECVNCGATSTPLWRRDGTGHYLCNACGLYYKMNGQNRPLIKPKRRL
ZF2: (Chr3R:3978980-3979123)
TLQSLQSAAKRAGTSCANCKTTTTTLWRRNASGEPVCNACGLYYKLHNV
3'CD: (Chr3R:3975269-3975455)
NRPLTMKKEGIQTRNRKLSSKSKKKKGLGGGCLPIGGHLGMGDFKPLDPSKGFGGGFSASM
3'CDS_1: (Chr3R:3974992-3975179)
AQHGHLSSGLHPAHAHMHGSWYTGGMGALGASSGLQGGFSTAGSLSGAVVPHSQPYHLGLSSM
3'CDS_2 (Chr3R:3974660-3974686)

Caenorhabditis elegans **(nematode)**
(classified according to expression patterns and function)

GATA1/2/3-like (expression in ecto- and neurectodermal derivatives)

>ELT-1 (NM_001038346.1) on ChrIV:9615133..9620113 (NC_003282.4)
MHYRDANYSISRNKVNLHHEEFMLDGVVGGEDHDMQNTNEVRAELDSLLRLDPDTNSIIDALHISQPIEQ
ENMDYEGKPVEFTLGTSSGGASLAPTSSTTAASIAPFSYNTSATNYYNTTPSSYPMFLNYQYAGGTTVTT
DMDAFSGMDMSMNNGVFGTQNNPSYFYPTTQLNTYGYDTLAAATTASGITVNNNQLNVNIVQGNGTIVPQ
PITQNIISTVSNVQSSVPINNSQPLTPTGLAGCSTSSGSSSASSSSANSTSTPKNTISKANRSSGGANNS
QFSTEDRECVNCGVHNTPLWRRDGSGNYLCNACGLYFKMNHHARPLVKPKKRQQNAQKRTGIECVNCRTN
TTTLWRRNGEGHPVCNACGLYFKLHKVERPITMKKDGIQTRNRKLSAKGSRRMKKENGGTPTSMGMPTTS
SSISSGIELDQSGVWGMKNTQPMLMTPTAYAFPASNFYFNSIEDQLEYKTCPPMMVDFGGQMKNLNLN

Comments: only embryonic expression. Mutant’s zygotic recessive lethal, makes no hypodermis, extra cell divisions in all hypodermal lineages, and extra neuron-like cells.

>ELT-3 NM_077920.3 on ChrX:13938663..13941124 (NC_003284.6)
MKDSQLSVKPAAYYCSPTMNDYRVEKVANTLLDPYVQLDQPTYADFTNAQVLNHQQEMLQMNFPTPLSTS
YMNTAQVTQTHQMPFNIFELNLSNFATFQPACDTPLPLLNSSPTHPYTTMSNFTPPPQDPLVAEPKPMKK
RMAAVQCHQNSICSNCKTRETTLWRRNGEGGVECNACNLYFRKNNRKRPLSLRKDGIMKRNRRPRNESPN
SAIRNTHQRHGHAAAC

Comments: expressed in hypodermal cells at ~ 240 min after first cleavage, later expressed in pharyngeal intestinal valve.

ELT-5/ELT-6 cluster at 1902-1917K on chromosome IV (NC_003282.4)

>ELT-5/egl-18 AAK3716 on ChrIV:1902322..1917826
MSISIMTETRPESAEQQHHEVLQRPSDEPCSGCKQLQKDVAKTISMVMERMDKLQYRLDELLKENNELKS
SSVSSGKASPSPAESRSSPKLVETVVAPVSGARKRKPKERSPPAAASPLPDFSNLMNGFMFDPLNMSNPN
GMMQLLSMVQQQQQQQHHQHIENQQSVSPPQSKSVKIEDPMDQDVKQEESERSDIPTATEAQNLLDALT
AQFSSNGQATSTTSPPSSSSQVQAVIEAVATPSSQSQDSSMFEKTETSGDPNAARCSNCRTDKTTAWRRD
AEGKLVCNPCGLYYRLHKVRRPIEMRKNHIQQRYRRKNKEKESSAATQIFNQLLTQMPTMATGGVSTDGA
INTFNLLEQISQFTQAQELMNSSATF

Comments: expressed at 28 cell stage in all grand-daughters of MS and AB founder cells, later expression most pronounced in seam cells, and later expression in various nervous and/or support type cells;

>ELT-6 AF353303_1 on ChrIV:1917853..1921616
MTSSKEEMPDEMVSREVEEEMEDRVEETATTAAACTGCAQLHKDIRQDVSKMMSKIDEVCGRLEALISEK
EKVLLEQMSESGSEEKYPPSPTESRASPSSVGKSNGGCRKRKPTKESVNRHLENGGSDSPFEKITRNIST
PVSASSPFPDFQNFNGFVFDPMANPQNMMNLLNLVQQQQHQAAAHHHAQQQAREQKPVKAEESKQQEPSE
NRNQSPSASVEQTLLDQLSMQFNGKSPSPTVHASTAAAAGSSEDDTSANSSISKCSNCSTIKTTAWRRDL
EGKLVCNACGLYYRLHRTHRPVHMRKDFIQQRFRRRMREDENPATSQAAVFSQLLGLPSMANGGANALTF
LEQINQLNQSQEQRKSP

Comments: Similar to ELT-5, but only very weakly expressed in MS and AB progeny, while having very strong expression within nervous system.

GATA4/5/6-like (expression in endo- and mesodermal derivatives)

>ELT-7 (AAC17756) on ChrV:4770485-4772903 (NC_003284.6)
MLPETTTLQPLPSVTTIMNEPPARSPMEDYYLFNQFQYNQNPYVYPPQPVYYNTWQDASTHHFDPFQSYQI
PSPYEQPIQSSMCTTPLQPLEDSRIIFDESLTKNENEQKSFVEQDSSYESSGNRFGSQKGKKIAKVIRDAC
CSHCSTTTTTLWRKNDEGNLECNACNLYYRHNKVKRPLSLCKQKPTTRKRRQAKKE

Comments: Expressed in intestine in larva and adult, required with ELT-7 in intestinal differentiation

END-1/END-3 cluster 27,701 bp apart on chr V (NC_003284.6)

>END-1 (AAB97516) on ChrV:13984943-13986806 (NC_003284.6)
MYQSSSSSPSPHLPYTYGVIDKDGNVHSHEMHFPDNHGNPSPLDATQMYQQAPVQYGGGDQYFDDFGQYFV
QSYDPAQQSTPVHPMFGSLDMMNCYSQQYPQIGQDYQQQEIENIPPVSTNRKIVNKKPSTFHTNSVCSNPN
CRTRETTLWRRTDSGAIECNGCSLYFRKNGIQRPAELCRKTIMKRNRRPRAEVQSPTPEDSKLCHNTTPLQ
NIPSQHFS

Comments: mRNA expressed in early E cell at 8 cell stage, low around 100 cell stage; sufficient to initiate endoderm differentiation.

>END-3 (NP_506490) on ChrV:14014507-14015656 (NC_003284.6)
MYSNSFSSSSSSSNSPMSFDFGFPQFPEQVQFNEEGYGTPSPDVLQNMNYHHYPAQDMTTNSYNGGYDNSM
QQNFMQPDNGAGYYNENYQQMPDFQFPVQNFDFTNQFEFTTPINDLQQSQTSINHTNPDNNENSMPEIPID
GGFNFFPAQEVQEWKPARKASKNKIKKISTMHINSSCSNCGCRETKLWRRNEQGETECNPCNLYERVKGHK
RPQHLWNKPAAKRRRRPVAPLVDSNAFNF

Comments: expressed in early E lineage; helps to specify mesendodermal lineages

ELT4/ELT-2 cluster 5336 bp apart on chr X (NC_003284.6)

>ELT-4 (NP_741888) on ChrX:10475456-10475834 (NC_003284.6)
MDNNYLDASHRKRLVCSNCNGTNTTLWRRKAEGDPVCNACGLYFKLHHVTRPIPMKKNKKHAVLPAPGISK
L

Comments: Expression in intestine at all stages of development, later pharyngeal expression
nonspeciific RNA phenotype, 1 allele (ca16) and strain (JM124) not characterized)

>ELT-2 (NP_509755) on ChrX:10481170-10483358 (NC_003284.6)
MDNNYNDNVNGWAEMEPSQPMGGLRLPTQNMDPPEQNNESQLSELPRMKIDNDYASPIERQSVITSGTNNY
EPKVETVTSFFHTGIDYSNFGMLDQTTMQPFYPLYSGIPVNTLGTFSGYTNSIYDKPSLYDPSIPTINIPS
TYPTVAPTYECVKCSQSCGAGMKAVNGGMMCVNCSTPKTTYSPPVAYSTSLGQPPILEIPSEQPTAKIAKQ
SSKKSSSSNRGSNGSASRRQGLVCSNCNGTNTTLWRRNAEGDPVCNACGLYFKLHHIPRPTSMKKEGALQT
RKRKSKSGDSSTPSTSRARERKFERASSSTEKAQRSSNRRAGSAKADRELSTAAVAAATATYVSHADLYPV
SSAAVTLPDQTYSNYYQWNTAATAGLMMVPNDQNYVYAATNYQTGLRPADNIQVHVMPVQDDETKAAARDL
EAVDGDS

Comments: Expression within endoderm at 2E stage, persists only in endoderm through adult; required along with ELT-7 for initiating and maintaining terminal differentiation of intestine

MED1/2 unlinked paralogs

>MED-1 (AF302237_1) on ChrX:12430531-12431055 (NC_003284.6)
MAYPYPVFNAENVFDNTQQQVGFYDYSTPFNGTYSFTTDYSYYNNYYDYVNTYASYYPTAMDSSSLNISST
TGSPNSSHFTTFTHFSTPSTSPSTSTQSSTTPSNSDNKKSFQCSNCSVTETIRWRNIRSKEGIQCNACFIY
QRKYNKTRPVTAVNKYQKRKLKVQETNGVDSF

Comments: mRNA accumulates in EMS nucleus, cytoplasmic in E and MS, and undetectable after, protein persists slightly longer
Immediate target of skn-1 to specify mesendoderm

>MED-2 (NP_498497) on ChrIII:6884441-6884965 (NC_003281.7)

Comments: mRNA accumulates in EMS nucleus, cytoplasmic in E and MS, as well as gonads of all adults
Immediate target of skn-1 to specify mesendoderm

Caenorhabditis briggsae **(nematode)**

GATA1/2/3-like

>**ELT-1** (CBG20056) on chrIV:9247640-9253069
MDYAHFDNFNSFEHHQEDKGSDSEIDAESEFNSLLRLDMDIHHIIDALQSDELSTMDYENNKSSSSVDFAL
ATSSGAVSSLVATSSASSAVAAATSSATAPFTAYNATPTANYYKDYNTSASTGYPMFLNYPYTTTTVAAGN
DMDFTNQDTMMHSGVFGVGAQNPNYFNPSIYQYGYDSLAAATSASGITVNNQVNVSIVQGSLPGTGSIVST
SSQIQPSATLLPRGAQGLTPTGINGCSTSSGSSSASSSSANSTSTPKTIGVPKPNRSAGANMGAGTEDREC
VNCGVHATPLWRRDGSGNYLCNACGLYFKMNHQARPLVKPKKRQQNAQKRTGIECVNCHTNNTTLWRRNGE
GHPVCNACGLYYKLHKVERPMAMKKEGIQTRNRKLSSKGQRRIKKENGDTTPTLGMSTASSSHTSGIELID
PTSVWGVKRETLPPMLMTTPATYSFPGSNFDWNSSSFVDQFQPVMVDFGGQLSKNLS

>**ELT-3** (CBG07341) on ChrX:19649087-19653028
MHRPHTCTDPYLCILCCICRHNSKTRSPPSNEYQYYQQEAVQPSHPARLPGISNFMKDSQISLKPATYYTA
AGSPTMNEYRVEKVAPALVEHPYIQLEQPTYADFTNAQVLSHQQEMLQMNFPTPLSTNYMNTAPVTQSQQV
PFNIFELISDSRLVSPNKHHQNFNTNLSNFTNFPQSETPLPLLNNSPPHSYSTMPNFSPPPQDPLVIEPKP
IKKRMAAVQCHQNSICSNCKTRETTLWRRNGEGGVECNACNLYFRKNNRKRPLSLRKDGIMKRNRRPRTES
PGATMRAHQRALAHQHAAAC

ELT5/6 at 15149-15153K of ChrIV

>**ELT-5/egl-18** (CBG13548) on chrIV:15149505-15152127
QNIIFQSMQMMAQRMDRMQNQLDELSKKPISTATSSVVSSGKVSPSPVSSPDRSVAAVVVASGASGARKRK
PKEHTPPTAASPLPDFTSFVNLFDPITMAANPNGMMHLLSLVQQQSQDGQHSAQQTPRRSMTPPEAKQVKS
EPKSVEEASTIENMKMEIVNEESSDMINAHTQNLLDALTAQFSTNLGGNNVATSSTASTVVQQVIEAVATP
SRSQDSSILDESTSSVVDPNAARCSNCHTDKTTAWRRDSEGKLVCNPCGLYYRLHKVRRPIEMRKNHIQQR
YRRKNKEKDVSASLTDQALLNQLFTQMPTMATGGSGAGGSPALSFLEQITQFTQAHELMNSSA

>**ELT-6** (CBG13549) on chrIV:15152797-15156803
MTSEEQDEVLEEKQKFEKTVKVEPEPKEEIGVESNGSSAVAATAEPAGPCAGCVQLHQEIRQDVKKIMNKI
ETVCERLEVMLAEKERMNQEQMSESGSEEKYAGSPSGSRESPAFQTNGKMLSAVMGNGGSRKRKPTKESVN
RLFENSLIHENGNGSTIEKVPRQVSTPVSASSPFADFNHFNGGLMFDPMPNPQNMMQFLNLVQQQQHHQQA
QQAAQIIQKEAQKVKEDVKQKPLEISNSPPNPSPNEQDLLSQLASQFNGKSPSPSVVHAAGSPEDDSSNSG
GSRCSNCSTTKTTAWRRDLAGKLVCNACGLYYRLHRTHRPVHMRKDFIQQRFRRKIKEDENPAISQEAVFS
QLLGMPPGGAAYSLMEHLNQLSQVQEESAKPSTGRTTESSKPDTGTTTPK

GATA4/5/6 like

>ELT-7 CBG08810 gene on chrV:1874685-1875944
MDHQHAEPLPSLSTVLAESSTRNQKEDLYLYDQFSNQSTFFNYPYPGQPVYYNPWQYSTLQPYDVYQYPML
TSYDQTIPVQPPIIDISTHSNVYEKPASVETAKTSSPESFMEHKTTMRRPAVKRTSSISRNCANCSTTETS
LWRKNEEGDLECK

END1/3 orthologs linked at 394-428K of ChrV

>END-1 CBG11413 on chrV:394638-396019
MYPISSSSSSASSSSSTPPLAPYTYGVMDKDGNIVAHEMLFSEDHGKPSPVHTPTMYEPTGSQYMMDPLYE
DFGQYFAVPPYVAPTAPVVPVNPMFGSFDMSMNQNYLQYPQFGQNYMAQENLVMQVKEKKSTGKSSVIRKP
STFHHNSVCSNPSCGTRQTTLWRRTDSGAIECNGCSLYFRKNGVQRPADLCNKQILKRNRRPRASPGVPVI
QVNTAVVHQEHNQTTEPMHAGRLL

>END-3a CBG11406 on chrV:425247-424323
MYSQSFASSSSSTNSNYTVPIDPMFHYQGFDMGYGTPSPGMPMDYNNMMTNQQFRSDSMGPPNDGMMFYGH
QQFPPMAEQFPMMDFQMMPEQAMDSGFQQNFQLGFQQDRFQPLPHQIPMAQSDIIHRQDSSLNSSPDVDFN
EVNGNFVNGMNGFPAPPVNDYNFSTTSPPPPKRKFTKKKVPKVSTMHLNTSCSNCGTRETKLWRRNEQGEP
ECNSCNLYERTKGVKRPATLWNKPTIKRRRRPVVAPVTQE

>END-3b CBG11404 on chrV:428272-429169
MYSQSFASSSSSTNSNYTVPIDPMFHSQGFDMGYGTPSPGIPMDYNNMMTNQQFRSDAMGPPNDGMMFYGH
QQFPPMAEQFPTMDFQMMPEQTMDSGFQQNFQFGFQQDQFQPHPIPMTQIHRQDSSSNSSPDVDFNEVNGN
FVNGMNGFPAPPVNDYNFSTTSPPPPKRKFTKKKVPKVSTMHLNTSCSNCGTRETKLWRRNEQGVPECNSC
NLYERTKGVKRPATLWNKPTIKRRRRPVVAPVTQE

C.briggsae has single ortholog to CeELT-2 and 4

>ELT-2/4 CBG1725 on chrX:6671190-6669503
MDTTYQENHNGWAEMESAQITQQSGGLRLPTQNMDPPAEQKDESQISELHRMKLENEYVPPIERQSVITNN
TMVYDGKIEPVAPQTMFYTGFDYPTTFGMLDPNGAIQNSYYNIYSIPVNNLNQPLINNFANPFSIQYFSIY
ETSVPTINIPTAYPAPTPVYECVKCSQNCGDGAKAVNGGMMCSNCAKVSEYPSPIVYPPSIGIPPVIEIPS
DQPPMKIPKASKKSSNANRGSNGSASRRQGLVCSNCNGTNTTLWRRNAEGDPVCNACGLYFKLHHIARPTS
MKKEGALQTRKRKSKTGEAVSPPVSRARERKYERSEKISRAATRRAGSAKAERELTTAAVAAATNPYAQAD
LYAIPSSTVGLQHQQDQTYSYYPWNPATTGIMMVPSDQNIYATTYNTGFVRPADVSAKLIASTSYVTAPVE
LLISLLSLLQVHVMPVQDDETKAAARDLEAVDNE

C.briggsae possesses 4 co-orthologs to the *C.elegans* MED1/2 genes

>MED CBG13715 chrIV:1010995-1008539
MEDGLENTMRMSDHKLSVFETSYCIQPVNNLDGIQYCCIPQRPRDQLSYFMVFDEFIMILKSHEAKMNDTE
YTERSDKEIRQNEAKPEIKSSIDGPQAQYPILQMMPAMMFVGTNAVTLPNPIANLQMDLMNQIKIDLMSLA
MSSPLFWNFPDLAATQNMEQQYPTPIWTPHDTVPINQEASEEKEEDVKENEAPQFTNFPANTRSMCQLDQN
EPTPSKSESKQCSNCSITKSCQWRNVTSSEGILCNACFVYERKYKKSRPMKAIQHYKKRTNDFPSSPATKL
AQGPSTPCKTPATRLAMESLSSFPATQFASATSTPKRRAMDPLSLLASPTPATQSYTEALPTSATQLVQDP
ATSTPKRRAMDPLSLLASPTPGTVAQAPTTPRMSRPSPATQLAMEASPTPSKLVQDPTTPVAPNSATPKRS
YRRKLLEESQKCSNCSIINSCRWRNVKSKESTLCNACFVYRRYIKKDRPTSAIESYKSRINEM

>MED CBG15489 on chrX:15669374-15669955
MNSQLAPISTGSVPDTPIPYSYADAHYWNSGNLNWNYLNTQATDYSDYTLYSNYYAYYYAQFFQSLSSNVS
TPSPTQIPQREPATMLPPLNFDFNATTPNPSSPSTSSTLAPMQLSAEMPIQSLTTVTCESTSRRDSKTRQC
TNCFVTKTSLWRNVTSSEGILCTACFTYKRKYKKNRPTKAIERYMRRNRNL

Two Linked Med genes at 7617K-7619K on ChrII

>MED CBG18154 gene on chrIII:7617458-7616847
MDFQFPTVSTASFLDTSSVPSTSSWDSGVVNRSTTYLDTQSINYSDYSYSSNYYANYYASFLQCLNSNTST
ATVVPSPTATSQTSMDLSWNGFQNNFNVSSSSISSSPSSSSTGFTPFSANSPTMPTPASTLSPTGSTTPRN
AKTYTKQCSNCFITESCQWRNVTSREGMLCNACFTYRRKYKKNRPTAAIEKYRCQKREKQL

>MED CBG18155 gene on chrIII:7618795-7619406
MNFQFPTFSTASFLDTSSVPSTSSWDSGVVNRSTTYLDTQSTNYSDYSYYSNYYANYYASFLQCLNSNTST
ATVVPSPTATSQTSMDLSWNGFQNNFNVSSSSISSSPSSSSTGFTPFSANSPTMPTPAFTLSPTGSTTPRI
AKTYTKQCSNCFITESCQWRNVTSREGMLCNACFTYRRKYKKNRPTAAIEKYRCQKREKQL

Helobdella robusta **(annelid, leech)**

>e_gw1.3.1105.1 [Helro1:66090] Helro1/scaffold_3:7650093-7651081
DGRECVNCGATSTPLWRRDGGGNYLCNACGLYYKMNGHNRPLVKPRRKHNSSRRIGTKCSNCSTISTTLW
RRNIKGEPVCNACGLYYKLHSINRPITMKKECIQTRNRKLTTK

>gw2.5.770.1 [Helro1:143547] Helro1/scaffold_5:3850156-3854554
GRECVNCGATSTPLWRRDGNGQYLCNACGLYHKINGQNRPLIKPKRRLSSARRAGTTCANCSTNMTTLWR
RNQNGDAVCNACGLYYKLHNVNRPITMKKEGIQTRNRKLSSKGKRHRRSGD

>e_gw1.5.630.1 [Helro1:67904] Helro1/scaffold_5:5822214-5822495
MWRRDSEGRCICNACGLYERANNGQKRNLRQARGKAPYNRPNQVCSNCSTRSTTMWRKTENGEVVCNACG
LYYKLYQKHRPLELKREKIQTRKR

Comment: Has very similar beginning to e_gw1.5.1164.1 [Helro1:68470], overlaps completely with end of gw2.5.861.1

>gw2.5.805.1 [Helro1:145048] Helro1/scaffold_5:5829784-5830125
CSNCGTTSTPMWRRDSEGRCICNACGLYERANNGQKRSLRQARGKAPYKRPNQVCSNCSTRSTTMWRKTE
NGEVVCNACGLYYKLYQKHRPLELKREKIQTRKRPLKKMKTRNE

Comment: Very similar to gw2.5.807.1

>gw2.5.861.1 [Helro1:147055] Helro1/scaffold_5:5836163-5836480
RACSNCGTTSTPMWRRDSEGRCICNACGLYERANNGQKRSLRQARGKAPYKRPNQVCSNCSTRSTTMWRK
TKNGEVVCNACGLYYKSYQKHRPLELKREKIQTRKR

>e_gw1.5.1164.1 [Helro1:68470] Helro1/scaffold_5:5838978-5839358
MWRRDSEGRCICNACGLYERANNGQKRSLRQTRGKAPYKRPNQVCSNCSTRSTTMWRKTENGEEVCNACG
LYYKLYQKHRPLELKRWLDDFFRMKFLQFKRSYKLLQIILKNELVVFLINLTYIP

>gw2.5.807.1 [Helro1:145054] Helro1/scaffold_5:5840862-5841203
CSNCGTTSTPMWRRDSEGRCICNACGLYEKANNGQKRSLRQVRGKAPYKRPNQVCSNCSIRSTTMWRKTE
NGEVVCNACGLYYKLYQKHRPLELKREKIQTRKRPLKKMKTRNE

>e_gw1.23.272.1 [Helro1:78647] Helro1/scaffold_23:2435508-2436203
CENCGTTKTTAWRRSESKELLCNACGCYYKLHKVHRPLHMAHNEIRSRTRRVNRSMALTSSAMMSSLDAG
NLLHDNGQQLIGEHLPGGNQQIVTS*

>e_gw1.28.188.1 [Helro1:80803] Helro1/scaffold_28:458165-459430
MNVFFADDLADDYFGDRKECVNCGAMGTPLWRRDGTGHYLCNACGLYQKMNGLNRPLTRQPKRLQTARRF
GLSCTNCQTVTTTLWRRSNEGEPVCNACGLYFKLHGIPRPLSMRKEGIQTRKRKPKSPGKSMNSADHKGY
LHNLYER*

>e_gw1.33.285.1 [Helro1:82644] Helro1/scaffold_33:553718-555407
MNIKKITNFSTILPNEFSNFSPNFPKECVNCGSPSTPLWRRDGVGHYLCNACGLYQKINGSNRPIVKQDT
FKRTNNKKLGATCSNCRTAVTTLWRRNNDGDVVCNACGLYYKLHGVRRPSVMRKDGIQTRKRKPRN

>gw2.36.90.1 [Helro1:128502] Helro1/scaffold_36:1760473-1761362
RKKGSVCSNCGSAASTLWRRNMLGETVCNACGLYHKLHGINRPMTMRKDAIQTRKRR

>e_gw1.1564.3.1 [Helro1:92960] Helro1/scaffold_1564:875-1141
NDLRECVNCAVKITPLWRRDCNGNYLCNACGLYYKMNGHSRPLVKPKRRLVSYSFIHSLARSFVHLFIHS
FIHSFIHSFIHSFVYLFV*

>e_gw1.3021.1.1 [Helro1:93034] Helro1/scaffold_3021:346-1101
RNSGLKCANCQTNLTTLWRRNKNGEPVCNACGLYYKLHNISRPITMKKDGIQSRNRKSKSTERKLKR
