# Supplementary figures and images for: The evolution of protostome GATA factors: Molecular phylogenetics, synteny, and intron/exon structure reveal orthologous relationships
Source: BMC Evol Biol. 2008 Apr 15;8:112. doi: 10.1186/1471-2148-8-112 (PMC2383905; doi:10.1186/1471-2148-8-112)

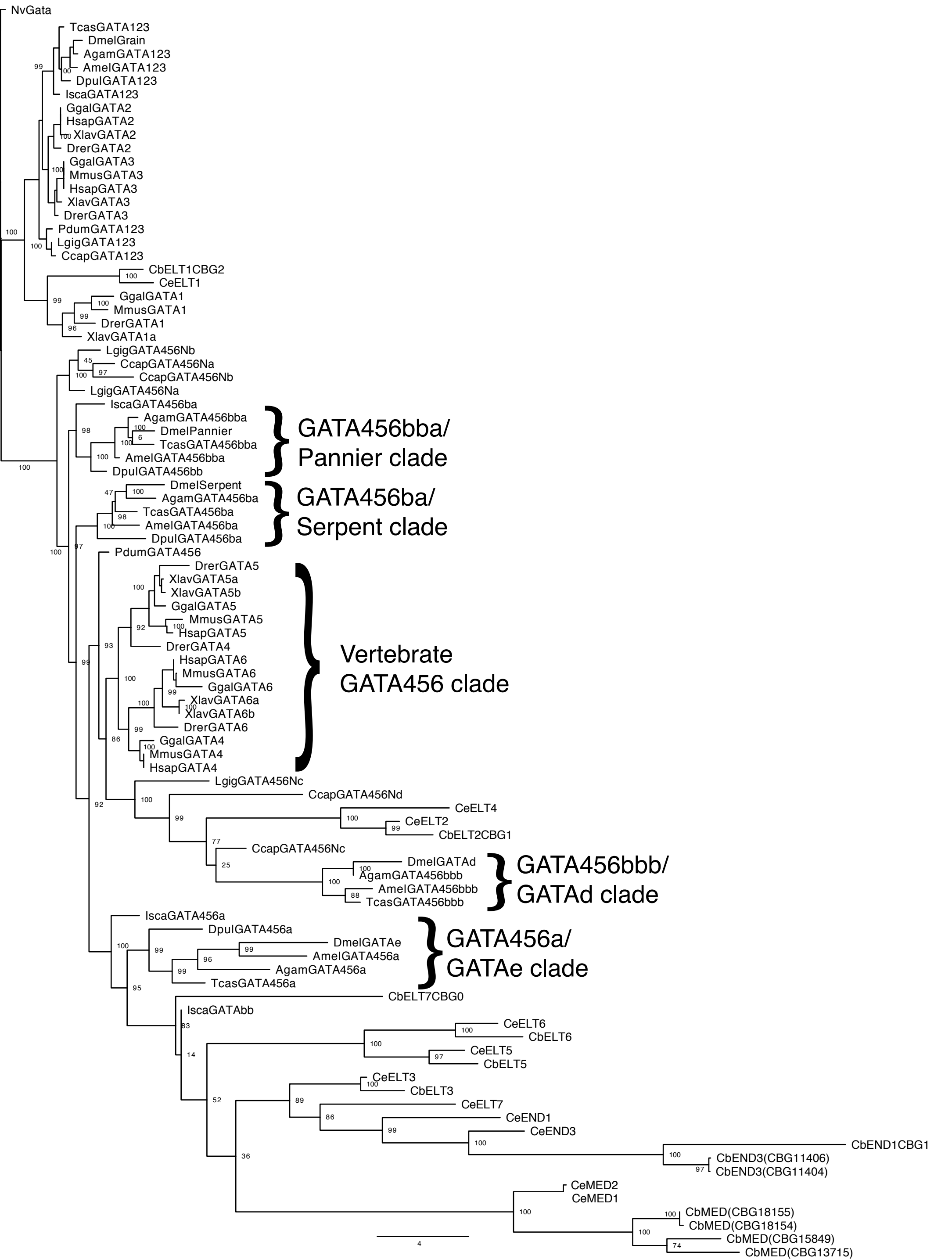

Supplement: Additional file 2 — ML analysis of conserved GATA dual-zinc finger domains of protostomes, including nematodes. This tree represents a Maximum Likelihood phylogenetic analysis of the conserved GATA dual-zinc finger domains including nematodes. [file 1471-2148-8-112-S2.pdf]
