# Supplementary material for: The evolution of protostome GATA factors: Molecular phylogenetics, synteny, and intron/exon structure reveal orthologous relationships
Source: BMC Evol Biol. 2008 Apr 15;8:112. doi: 10.1186/1471-2148-8-112 (PMC2383905; doi:10.1186/1471-2148-8-112)
Supplement: Additional file 3 — Signature Amino Acids for GATA orthologs. This file contains an annotated alignment of the consensus sequences for GATA conserved domains from various orthology groups. [file 1471-2148-8-112-S3.pdf]

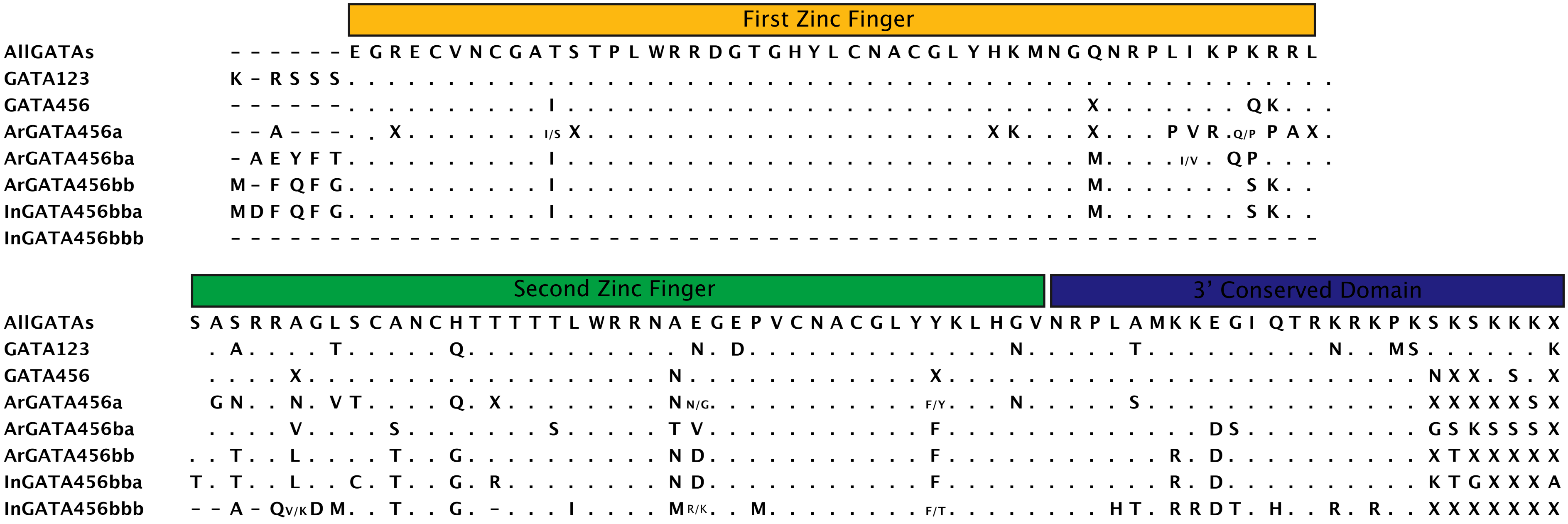

Additional File 4: Alignment of consensus sequences for GATA conserved domains from various orthology groups (GATA123-Bilaterian GATA123,GATA456-Bilaterian GATA456, ArGATA456a-Arthropod GATA456a, ArGATA456ba-Arthropod GATA456ba, ArGATA456bb-Arthropod GATA456bb, InGAT456bba-Insect GATA456bba, InGATA456bbb-Insect GATA456bbb). The colored boxes indicate the ancestral exon regions of the alignment.
